# Starvation tactics using natural compounds for advanced cancers: pharmacodynamics, clinical efficacy, and predictive biomarkers

**DOI:** 10.1002/cam4.1467

**Published:** 2018-05-06

**Authors:** Khalid El Bairi, Mariam Amrani, Said Afqir

**Affiliations:** ^1^ Faculty of Medicine and Pharmacy Mohamed Ist University Oujda Morocco; ^2^ Equipe de Recherche en Virologie et Onco‐biologie Faculty of Medicine Pathology Department National Institute of Oncology Université Mohamed V Rabat Morocco; ^3^ Department of Medical Oncology Mohamed VI University Hospital Oujda Morocco

**Keywords:** advanced cancer, angiogenesis, clinical trials, drug discovery, natural compounds, predictive biomarkers, vascular disrupting agents

## Abstract

The high mortality associated with oncological diseases is mostly due to tumors in advanced stages, and their management is a major challenge in modern oncology. Angiogenesis is a defined hallmark of cancer and predisposes to metastatic invasion and dissemination and is therefore an important druggable target for cancer drug discovery. Recently, because of drug resistance and poor prognosis, new anticancer drugs from natural sources targeting tumor vessels have attracted more attention and have been used in several randomized and controlled clinical trials as therapeutic options. Here, we outline and discuss potential natural compounds as salvage treatment for advanced cancers from recent and ongoing clinical trials and real‐world studies. We also discuss predictive biomarkers for patients' selection to optimize the use of these potential anticancer drugs.

## Introduction

**Figure 1:**
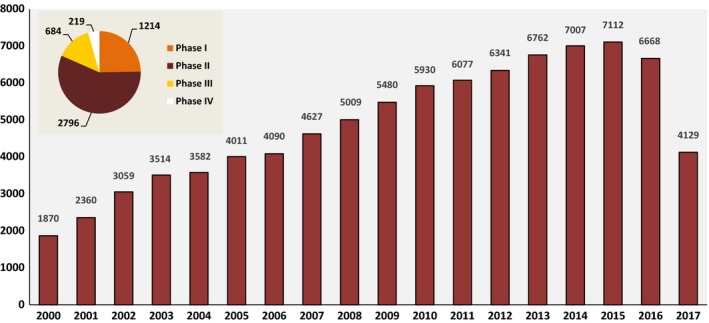
Evolution of published PubMed‐indexed articles from 2000 to 2017 and clinical trials related to angiogenesis research. Data for this figure were extracted from PubMed/Medline and ClinicalTrials.gov [accessed 12 September 2017 and analyzed by Excel (Microsoft Office 2007™)].

Management of some advanced and metastatic cancers is a challenging area in modern oncology because of poor prognosis, drug resistance development, and few available therapeutic options beyond first line. These obstacles have prompted clinical investigation for novel anticancer compounds, such as those from natural sources, targeting the proposed model of cancer hallmarks. Tumor angiogenesis is still the major targeted and most important cancer hallmark allowing for tumor cell invasion 1, 2, 3. It has recently gained more interest in clinical development of anticancer drugs with more than 5000 clinical trials recorded on the U.S. National Institutes of Health database (http://www.clinicaltrials.gov) as well as more than 94,000 articles according to the *PubMed/Medline* database (Fig. 1). In addition, tumor angiogenesis and lymphangiogenesis have become an important research area over the last decades for their crucial role as an essential component for the growth and spread of cancer cells 3, 4. Angiogenesis‐mediated metastatic spread through the bloodstream worsens the prognosis of most aggressive solid tumors 5, 6, 7, 8. A large amount of literature has shown the link between tumor angiogenesis, metastasis, and overall survival (OS) in patients with advanced cancer 9, 10. Of note, blood vessels of tumors differ from normal resting blood vessels; this difference makes tumor blood vessels a reliable target for tumor‐directed treatment. Classically, vascular‐targeted therapeutic agents may act by neutralizing the angiogenic proteins, inhibiting their synthesis by cancer cells, directly inducing endothelial cell (EC) apoptosis, inhibiting endothelial receptors of angiogenic proteins 11, 12, or by disrupting tubulin network of the established tumor vasculature and therefore tumor blood flow interruption 13, 14, 15, 16, 17. The nutritional supply of tumor tissues is under the regulation of various cytokines that (1) increases the permeability and the widening of tumor blood vessels, (2) stimulates proteolytic enzymes and cellular infiltration, (3) rebuilds blood vessels, and (4) protects ECs from apoptosis. Hypoxia increases the production of proangiogenic factors by tumor cells, macrophages, and other immune cells from the tumor microenvironment 18, 19, 20. Targeting tumor vessels with natural compounds such as trabectedin, plitidepsin, and combretastatins, which induce tumor starvation, has gained space and interest in cancer drug development for advanced cancers 21, 22, 23, 24, 25, 26. These compounds showed significant improvement in clinical outcomes and might become an alternative therapeutic for these challenging cancers according to recent human clinical trials. This critical review analyzes the clinical relevance of using natural compounds targeting tumor vessels alone or in combination with other anticancer drugs with a focus on recent clinical trials as well as the importance of predictive biomarkers for their optimal use in the management of advanced and metastatic cancers.

## Targeting the Tumor Vasculature by Natural Compounds: Perspectives from Clinical Trials

**Figure 2:**
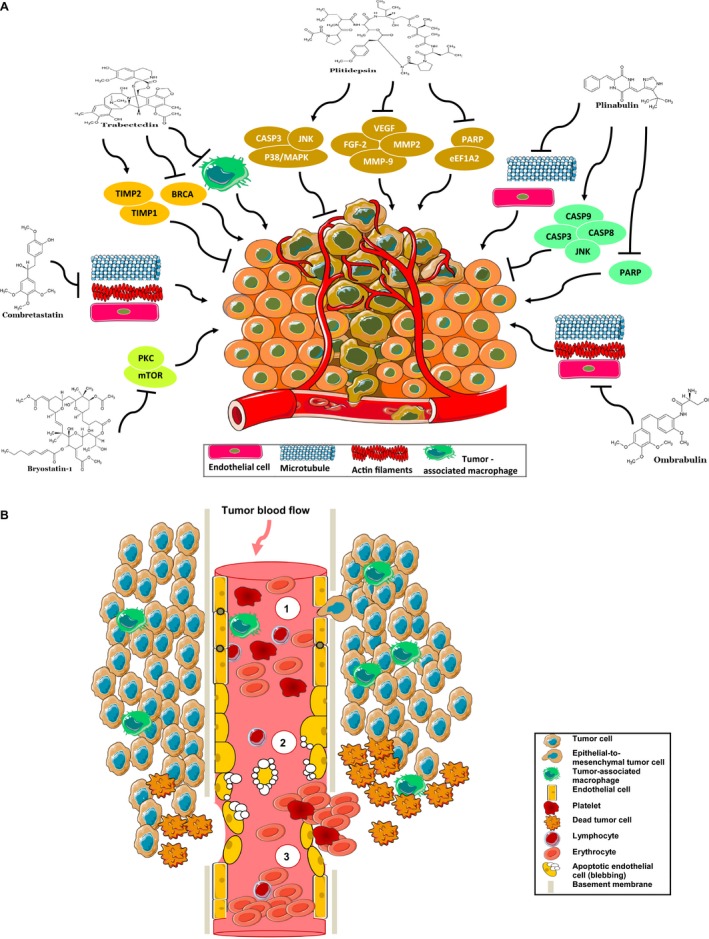
(A) Mechanisms of vascular shutdown and signaling pathways targeted by antivascular natural compounds (for details, see main text). BRCA, breast cancer susceptibility gene; CASP3, caspase‐3; CASP8, caspase‐8; CASP9, caspase‐9; eEF1A2, eukaryotic elongation factor 1A2; FGF‐2, fibroblast growth factor 2; JNK, c‐Jun N‐terminal kinase; MMP‐9, matrix metalloproteinase 9; mTOR, mammalian target of rapamycin; p38/MAPK, mitogen‐activated protein kinase; PARP, poly(ADP‐ribose) polymerase; PKC, protein kinase C; TIMP1, tissue inhibitor of metalloproteinase 1; TIMP2, tissue inhibitor of metalloproteinase 2; VEGF, vascular endothelial growth factor. (B) Cellular mechanisms of tumor vascular disruption by natural compounds. (1) Normal tumor blood flow. (2) Reduced tumor blood supply: Alteration of the cytoskeleton and disruption of cell–cell adhesion molecules cause impairment, morphology changes, and blebbing of endothelial cells, and therefore an increase in vascular permeability (protein extravasation, para and transcellular permeability). (3) Vasoconstriction and shutdown of the established tumor vessels: after blebbing; endothelial cells die by apoptosis, and rapid collapse of tumor vessels is observed (for details, see reviews by Jaroch et al. 35, Chase et al. 36, and Tozer et al. 37).

Natural compounds are a major source of small molecular weight angiogenesis inhibitors 23, 27. Considering these perspectives, there have been extensive studies on natural compounds that showed potent and promising antiangiogenic activity as well as destabilizing microtubule dynamics (an important player in tumor vasculature) 28, 29, 30. These compounds act through multiple cell‐signaling pathways and decrease the development of resistance by cancer cells 31, 32, 33, 34. A number of compounds derived from natural sources were screened in order to study their potential as antiangiogenesis inhibitors (Fig. 2). Published clinical trials and real‐world studies showed promising results in the advanced setting using natural compounds as a single agent or in combination with other anticancer drugs.

### Trabectedin (ET‐743)

Trabectedin (Yondelis^®^; PharmaMar^®^) is an alkaloid originally derived from *Ecteinascidia turbinata* (a Caribbean tunicate) and one of the most successful marketed natural drugs for relapsed ovarian cancer (OC) and soft‐tissue sarcoma (STS) 22, 38, 39, 40. Trabectedin has been shown to have antiangiogenic properties in several preclinical studies by activation of TIMP1 (tissue inhibitor of metalloproteinase (1) and TIMP2 (tissue inhibitor of metalloproteinase (2) as well as exerting multimodal targeting of other cancer signaling pathways 41, 42, 43. Recently, in a neoadjuvant phase II trial for myxoid liposarcoma, trabectedin exhibited a decrease in the arborizing tumor vascularity 44. Furthermore, trabectedin was found to generate DNA damage, strong inhibition of transcription, and cytokine production related to tumor‐associated macrophages (TAMs) which are involved in cell survival, migration, and metastasis 22, 45, 46, 47. Trabectedin has also been shown to alter the DNA repair genes including *BRCA1* (breast cancer type 1 susceptibility gene) and *BRCA2* (breast cancer type 2 susceptibility gene), which launches new perspectives as a chemotherapy option for patients with mutated ovarian and breast cancer (BC) 48. More than 70 clinical trials registered in the US National Institutes of Health database are currently ongoing evaluating trabectedin in phase I/III studies especially for STS and OC (Fig. S1).

#### Phase I trials

As a single agent, trabectedin has been used in various schedules to determine the safety, tolerability, maximum tolerated dose, and recommended doses for phase II studies. In this direction, sequential cohorts of patients with advanced solid tumors were enrolled and treated with doses ranging from 0.46 to 0.80 mg/m^2^ (1‐h schedule) and 0.30 to 0.65 mg/m^2^ (3‐h schedule) 49. The most observed side events in these two schedules included neutropenia, fatigue, and transaminase elevation, which translates into an acceptable safety profile. Furthermore, maximum tolerated dose for trabectedin given weekly was 0.58 mg/m^2^ as a 3‐h infusion and 0.61 mg/m^2^ as a 1‐h infusion 49. This safety profile was confirmed in another phase I study enrolling pediatric patients with refractory and relapsed solid malignancies 50. In this study, recommended dose of trabectedin in children with advanced solid tumors was 1.5 mg/m^2^ given intravenously over 24 h every 3 weeks (q3 wk). Trabectedin was combined with dexamethasone and granulocyte colony‐stimulating factor (G‐CSF) support to prevent hepatotoxicity and neutropenia 50. In Japanese patients with STS, trabectedin was given at a starting dose of 0.9 mg/m^2^ as a 24‐h continuous infusion q3 wk. This dose was escalated to 1.2 mg/m^2^ and then to 1.5 mg/m^2^ using a “3 + 3” design 51. Frequent grade 3 or 4 adverse events including elevation of aminotransferase and neutropenia were observed. In this cohort, the recommended dose for evaluating trabectedin in phase II studies for tissue sarcoma was 1.2 mg/m^2^ 51.

Combination of trabectedin with other anticancer drugs, namely gemcitabine, capecitabine, carboplatin, paclitaxel, and doxorubicin, showed feasible administration, manageable toxicities (including neutropenia, thrombocytopenia, and hepatotoxicity as frequent adverse events), and preliminary antitumor activity in a wide spectrum of advanced solid tumors, which was previously reported in single‐arm trials 52, 53, 54, 55, 56, 57. Recently, Sessa et al. 58 evaluated cisplatin at a fixed dose of 75 mg/m^2^ 1‐h infusion followed by a dose‐escalation schedule using trabectedin 3‐h infusion both administered on day 1 q3 wk in patients with advanced cancers. This combination was well tolerated with reversible side events and concordant with known toxicities of trabectedin. Based on this, trabectedin at 0.60 mg/m^2^ was declared the recommended dose for further phase II investigations 58. Furthermore, data from an open‐label phase I trial investigating docetaxel (60 or 75 mg/m^2^; 1‐h intravenous infusion) given q3 wk followed by escalating doses of trabectedin (0.4–1.3 mg/m^2^ by 3‐h intravenous infusion) showed a favorable toxicity profile and no evidence of drug–drug interactions 59. In conclusion, trabectedin as single agent or in combination is well tolerated, which merits further evaluation in randomized phase II‐III trials. Serious adverse events such as neutropenia and transaminase elevation can be prevented by concomitant use of prophylactic G‐CSF and corticosteroids.

#### Phase II trials

Moving from these encouraging evidences from phase I trials, trabectedin was used in numerous phase II trials to assess its preliminary efficacy in STS, translocation‐related sarcoma, OC, BC, and other advanced solid tumors.

##### Soft‐tissue sarcoma

In STS, which represents a very heterogeneous cancer, initial nonrandomized evaluation of trabectedin demonstrated durable objective responses and relatively high survival rate in previously treated patients with recurrent or metastatic STS with disease progression despite prior conventional therapy (doxorubicin and ifosfamide) 60, 61, 62. After these favorable data, Demetri et al. 63 randomized 270 patients with unresectable or metastatic liposarcoma or leiomyosarcoma after failure of anthracycline‐based chemotherapy. This study assigned patients to one of two trabectedin regimens including 1.5 mg/m^2^ 24‐h intravenous infusion once q3 wk versus 0.58 mg/m^2^ 3‐h intravenous infusion every week for 3 weeks 63. A documented superiority in disease control was seen with the q3 wk 24‐h schedule compared to 3 h every week for 3 weeks including improved median progression‐free survival (PFS) (3.3 months vs. 2.3 months; HR: 0.755; 95% CI: 0.574–0.992; *P *=* *0.0418) and median OS (13.9 months vs. 11.8 months; HR: 0.843; 95% CI: 0.653–1.090; *P *=* *0.1920), respectively 63. Based on these results, trabectedin was then approved in Europe by the European Medicines Agency (EMA) and in other parts of the world and recently in the United States for treating patients with advanced sarcoma after failure of standard therapy based on ifosfamide and doxorubicin 64, 65. Combination therapy with dexamethasone was found to ameliorate the safety of trabectedin by reducing the myelosuppression and hepatotoxicity in patients with recurrent advanced STS, and it is now required in all trabectedin clinical trials 66. In an open‐label randomized and controlled phase II study of the Spanish Group for Research on Sarcoma (GEIS), Martin‐Broto et al. 67 compared trabectedin 1.1 mg/m^2^ plus doxorubicin 60 mg/m^2^ with doxorubicin alone at a dose of 75 mg/m^2^. PFS was the primary endpoint; results did not show superiority of the combination. However, this exploratory translational study found that *FAS* (fas cell surface death receptor, also known as CD95) and p53 (phosphoprotein p53) are potent prognostic biomarkers for trabectedin evaluation which warrant more examination in the future clinical trials 67. Similarly, according to the TRUSTS trial, first‐line trabectedin did not show superiority compared to doxorubicin which is still the standard treatment of metastatic STS 68. In addition to these safety data, these phase II trials in STS have also provided important perspectives on predictive biomarkers. Italiano et al. 69 analyzed tumor tissue makers including *ERCC1* (excision repair cross‐complementation group 1), *ERCC5* (excision repair cross‐complementation group 5; also known as XPG), and *BRCA1* SNPs and their transcripts in 113 patients with advanced STS as well as their association with 6‐month PFS and OS based on log‐rank tests (Fig. S4). High expression of these three biomarkers was significantly associated with improved PFS and OS, which can be a potential signature for trabectedin response 69. For the moment, trabectedin is a promising approach in the second‐line setting of metastatic STS. Of note, trabectedin combined with doxorubicin showed also potent antitumor activity in advanced uterine or soft‐tissue leiomyosarcoma 70 and significantly reduced the risk of death and disease progression in advanced translocation‐related sarcoma 71.

##### Ovarian cancer

The SENDO trial inaugurated the use of trabectedin in women with OC that relapsed after platinum–taxane first‐line therapy 72. Trabectedin was administered at a dose of 1.3 mg/m^2^ as a 3‐h intravenous infusion q3 wk to 51 patients either sensitive (*n* = 29) or resistant (*n* = 30). Objective response rate was 43% and 7% in platinum‐sensitive patients and platinum‐resistant patients, respectively 72. Following these preliminary results, another phase II study was conducted to evaluate the activity of single‐agent trabectedin at 0.58 mg/m^2^ as a 3‐h infusion weekly every 4 weeks (q4 wk) in previously treated OC patients with platinum‐based chemotherapy (66 platinum‐sensitive and 81 platinum‐resistant) 73. In the sensitive cohort, in the 62 evaluable patients, the overall response rate (ORR) was 29% and the median PFS was 5.1 months. In the resistant/refractory cohort, 79 patients were evaluable and the ORR was 6.3% with a median PFS of 2.0 months 73. In summary, this trial found trabectedin effective in the platinum‐sensitive cohort of patients with OC. Later, to confirm these results in this platinum‐sensitive population, Del Campo et al. 74 randomized 108 patients to receive two dosing schedules of trabectedin at a dose of 1.5 mg/m^2^ over 24 h (n = 54; arm 1) or 1.3 mg/m^2^ over 3 h (*n* = 53; arm 2). In platinum‐sensitive and advanced OC, trabectedin was well tolerated and effective in both schedules—ORR was 38.9% in arm 1 and 35.8% in arm 2, and median time to progression (TTP) was 6.2 and 6.8 months for arm 1 and arm 2, respectively. In addition, most responses were seen in patients with serous histology (62.5%) and with large tumor size (<5 cm) (77.5%) 74. Del Campo et al. attempted to compare clinical activity in a pooled analysis enrolling these three phase II trials. The investigators demonstrated that the q3 wk regimen over 3 h is superior to the weekly schedule in terms of efficacy (median PFS: 5.6 vs. 2.8 months) 75. In this regard, a large randomized phase III trial showed interesting results for trabectedin as second‐line treatment for OC (see further). Considering the amount of evidence suggesting high response rate with trabectedin in OC patients with BRCAness phenotype (defined as the group of patients with documented repeated previous responses to platinum‐based therapy), a phase II study (MITO 15 trial) was conducted in this population 76. One hundred patients with recurrent *BRCA*‐mutated OC were enrolled in the trial and treated with 1.3 mg/m^2^ q3 wk over 3 h with trabectedin as single agent. As expected, platinum‐sensitive patients (*n* = 46) had better response compared to platinum‐resistant population (*n* = 48; ORR: 47.8% vs. 31.2%). A key problem in this study arose after finding that there was no statistically significant difference between patients considering the *BRCA* gene mutational status. The authors hypothesized that such unexpected results may be due to the small number of recruited patients as well as the possible presence of other genes related to the DNA repair system 76 (reviewed by Monk et al. 48). A definitive answer to this question may be provided by the recently initiated phase III trial that will include patients with recurrent OC and *BRCA*‐mutated and/or BRCAness phenotype (NCT02903004, MITO‐23).

##### Breast cancer

The first study investigating the activity of single‐agent trabectedin as an alternative therapy in advanced BC included patients who have previously received palliative therapy with taxanes or anthracyclines 77. Trabectedin was given to 27 patients at a dose of 1.5 mg/m^2^ over 24 h q3 wk. The ORR was 14%, three confirmed partial responses and one unconfirmed partial response were observed, and median TTP was 2.14 months 77. In similar conditions, Goldstein et al. 78 compared trabectedin 3‐h infusion at 1.3 mg/m^2^ q3 wk or 0.58 mg/m^2^ weekly for 3 of 4 weeks in a randomized multicenter phase II trial. Trabectedin modestly prolonged PFS and median TTP in the q3 wk arm compared with the weekly arm 78.

Data obtained from in vitro studies supported the use of trabectedin in *BRCA*‐mutated BC which is more sensitive to DNA‐damaging therapies 48, 79. Based on these pharmacogenomic findings, 38 patients with confirmed germline *BRCA*‐mutated metastatic BC were treated with trabectedin 1.3 mg/m^2^ over 3 h q3 wk 80. Median PFS was 3.9 months, and eight enrolled patients (21%) showed modifications in tumor volume. Unfortunately, the trial was closed due to low recruitment rate, and these results are immature 80. Remarkably, a further very recent subset analysis of a phase II trial found that trabectedin had a high antitumor activity in advanced BC patients with *BRCA2* mutations 81. In addition to *BRCA* genes, another phase II trial analyzed Xeroderma pigmentosum G gene (*XPG*), which is involved in the DNA repair, to predict the efficacy of trabectedin in advanced BC 82. In this study, 44 patients with hormone receptor‐positive, HER2‐negative, advanced BC were treated with trabectedin at a dose of 1.3 mg/m^2^ over 3‐h infusion q3 wk and stratified according to the *XPG* status (high *XPG* (>3) or low *XPG* (≤3)) 82. This schedule was found to have limited efficacy in this population (only one complete response in low‐*XPG* cohort), and the predictive value of the *XPG* expression was not noted. Therefore, the reason for the negative results when using gene signatures as prognostic and predictive tools in trabectedin efficacy is currently not completely understood, and large phase III trials are awaited. Promising data from these trials provided optimistic prospects in advanced BC with the currently available armamentarium salvage agents for this indication. Other phase II trials for other advanced tumors are listed in Table 1.

**Table 1:** Published phase II trials of trabectedin in other advanced cancers

Author (year)	Regimen and enrollment	Indication	Findings
Belli et al. (2016) 83 NCT01339754	Trabectedin 1.3 mg/m^2^ as a 3‐h continuous intravenous infusion every 3 weeks (q3 wk) (*n* = 25)	Metastatic pancreatic cancer	Median PFS and OS were 1.9 months and 5.2 months, respectively. Grade >2 neutropenia was seen in 44% of patients. Tissue analysis of 17 patients enrolled in this study identified 30 predictive genes associated with better prognosis. Single‐agent trabectedin had negligible antitumor activity as salvage therapy for advanced pancreatic adenocarcinoma
Michaelson et al. (2012) 84 NCT00147212	Cohort A: weekly 3‐h infusion at 0.58 mg/m^2^ for 3 of 4 weeks (*n* = 33). Cohort B1: 24‐h infusion at 1.5 mg/m^2^ q3 wk (*n* = 5). Cohort B2: 24‐h infusion at 1.2 mg/m^2^ (*n* = 20).	Metastatic castration‐resistant prostate cancer	Median TTP was 1.5 months in cohort A and 1.9 months in cohort B2. At 1.2 mg/m^2^ q3 wk and 0.58 mg/m^2^ weekly, nausea, fatigue, neutropenia, and transaminase elevation were the most observed side events. This two schedules showed modest activity in metastatic castration‐resistant prostate cancer
Blay et al. (2004) 85 NCT00003939	Trabectedin was given at 1.5 mg/m^2^ per cycle as a 24‐h infusion q3 wk (*n* = 28)	Advanced gastrointestinal stromal tumors (GIST)	Trabectedin at 1.5 mg/m^2^ is not effective in advanced GIST. Grades 3–4 neutropenia, thrombocytopenia and transaminase elevation were the most observed side events at this dose
McMeekin et al. (2009) 86 NCT00050440	Trabectedin as a 3‐h infusion q3 wk at a starting dose of 1.3 mg/m^2^ with dexamethasone pretreatment (*n* = 50)	Persistent or recurrent endometrial cancer (PREC)	Median TTP and PFS were both 1.8 months, and median OS was 6.7 months. Nausea, asthenia, vomiting, and transaminase elevation were the most frequent side events. Single‐agent trabectedin displayed minimal antitumor activity in this pretreated patients with PREC
Paz‐Ares et al. (2007) 87	Trabectedin over a 3‐h intravenous infusion q3 wk from 1650 μg/m^2^ to 1300 μg/m^2^ (*n* = 21).	Advanced colorectal cancer	This schedule is well tolerated in pretreated advanced colorectal cancer at 1.3 mg/m^2^. Trabectedin as single agent is not effective in metastatic colorectal cancer

PFS, progression‐free survival; OS, overall survival; TTP, time to progression.

#### Phase III trials

##### Ovarian cancer

Based on several experimental studies showing synergistic effects between doxorubicin and trabectedin and early favorable safety–efficacy studies 22, 52, 88, 89, a large randomized, controlled phase III trial (OVA‐301) was conducted to assess the efficacy of combining liposomal doxorubicin (PLD) given at 30 mg/m^2^ with trabectedin infused intravenously at a dose of 1.1 mg/m^2^ q3 wk (arm 1) versus PLD alone at 50 mg/m^2^ q4 wk (arm 2) 90. A total of 672 women were randomly allocated to the two arms: 329 received PLD alone and 334 received the combination. Patients were stratified according to platinum resistance, and those that discontinued treatment were excluded from the analysis. Importantly, in the arm containing trabectedin the ORR (35.3% vs. 22.6%) and median PFS (7.3 months vs. 5.8 months) were significantly improved. Furthermore, platinum‐sensitive patients had better PFS with the combination arm compared to PLD alone (9.2 months vs. 7.5 months). In contrast, this doublet did not show any benefit in the platinum‐resistant cohort, which is still a poor prognostic subgroup of patients with OC (see Fig. S5 for survival curves) 90. Additionally, an exploratory analysis was performed to evaluate whether 13 selected predictive biomarkers influenced clinical benefit 91. High expression of nibrin, which is involved in DNA double‐strand break repair, was found to be significantly associated with worse survival of patients with serous OC treated with trabectedin + PLD 91. At this time, trabectedin is not approved in the United States for treating OC in second line. Additional clinical trials including patients with platinum‐sensitive advanced or relapsed OC are undergoing, evaluating the combination of trabectedin with PLD versus carboplatin/PLD (phase III INOVATYON trial, NCT01379989) and combination of trabectedin with PLD versus PLD alone (phase III ORCHYD trial, NCT01846611).

##### Soft‐tissue sarcoma

In metastatic translocation‐related sarcomas, Blay et al. 92 conducted a multicenter, randomized phase III trial to evaluate first‐line trabectedin (arm 1; 1.5 mg/m^2^ 24 h q3 wk) versus doxorubicin/ifosfamide (arm 2; doxorubicin 75 mg/m^2^ q3 wk, or doxorubicin 60 mg/m^2^ plus ifosfamide (range: 6–9 g/m^2^) q3 wk). Sixty‐one patients were randomly assigned to arm 1 and 60 to arm 2, but only 88 were treated after pathology review confirmation. The primary endpoint was PFS. No statistically significant difference was observed in the two arms in terms of PFS and OS in this setting 92. In advanced liposarcoma or leiomyosarcoma, Demetri et al. 93 randomized 518 previously pretreated patients to receive trabectedin at a starting dose of 1.5 mg/m^2^ over 24 h (*n* = 345) versus dacarbazine at a starting dose of 1 g/m^2^ as a 20‐ to 120‐min infusion (*n* = 173). Interestingly, trabectedin reduced the death and risk of progression by 45% compared with dacarbazine (median PFS: 4.2 vs. 1.5 months). In addition, this trial demonstrated that trabectedin is superior in terms of disease control in comparison with standard dacarbazine in patients with advanced liposarcoma and leiomyosarcoma after failure of first‐line chemotherapy, despite nonstatistically significant OS benefit (13% reduction in risk of death; median OS: 12.4 vs. 12.9 months) 93. It should be noted that the positive results from this pivotal trial were the main reason for trabectedin approval in the United States by the US Food and Drug Administration (FDA) and in other parts of the world. Additionally, considering retrospective studies published until this time, trabectedin showed interesting efficacy in routine clinical practice 94, 95, 96, 97, 98. Moreover, from a medico‐economic point of view, trabectedin was found to be cost‐effective as an orphan drug for patients with short life expectancy 99. Overall, these data from trabectedin clinical trials (Table 2 and Table S1) are of potential clinical relevance as salvage therapy and promising drug in STS and OC.

**Table 2:** Published phase III trials of trabectedin

Author (year)	Regimen and enrollment	Indication	Response rate (RR)	Progression‐free survival (PFS)	Overall survival (OS)
Demetri et al. (2016) 93 NCT01343277	Arm 1: trabectedin (*n* = 345, 1.5 mg/m^2^ as a 24‐h infusion) Arm 2: dacarbazine (*n* = 173, 1 g/m^2^ as a 20‐ to 120‐min infusion)	Metastatic liposarcoma or leiomyosarcoma after failure of conventional chemotherapy	Trabectedin: 9.9% Dacarbazine: 6.9%	Trabectedin: 4.2 months Dacarbazine: 1.5 months	Trabectedin: 12.4 months Dacarbazine: 12.9 months
Blay et al. (2014) 92 NCT00796120	Arm 1: trabectedin (*n* = 61, 1.5 mg/m^2^, 24‐h infusion every 3 weeks (q3 wk) Arm 2: doxorubicin (*n* = 60, 75 mg/m^2^, q3 wk infusion, or doxorubicin 60 mg/m^2^ infusion plus ifosfamide (range: 6–9 g/m^2^) q3 wk infusion)	Translocation‐related sarcomas	Trabectedin: 5.9% Arm 2: 27.0%	Nonstatistically different between treatment arms	Not reached
Monk et al. (2010) 90 Monk et al. (2012) 100 NCT00113607	Arm 1: *n* = 337 PLD^a^ (30 mg/m^2^) + trabectedin (3‐h infusion of 1.1 mg/m^2^ q3 wk) Arm 2: PLD (*n* = 335, 50 mg/m^2^ q4 wk)	Relapsed ovarian cancer	Arm 1: 27.6% Arm 2: 18.8%	Arm 1: 7.3 months Arm 2: 5.8 months	Arm 1: 22.2 months Arm 2: 18.9 months

a Pegylated liposomal doxorubicin.

### Combretastatin

Combretastatin (CBT) is an antiangiogenic agent derived from *Combretum caffrum*, a South African bush willow 101. It belongs to the family of stilbenes, which possess antiangiogenic properties that cause a vascular shutdown in cancer tissues, leading to tumor necrosis by altering the cytoskeleton of ECs and, therefore, their proliferation and migration (Fig. S3) 35, 102. In this context, CBT is currently undergoing several clinical trials by Mateon Therapeutics^®^, Inc., NCI (National Cancer Institute), and the Leukemia and Lymphoma Society for leukemia, thyroid cancer, OC, gastrointestinal cancers, and other solid tumors (Table S2). CBT showed excellent antiangiogenic activity in preclinical studies by interfering with microtubule dynamics 103, 104, 105.

Several clinical trials have shown CBT clinical safety in different tumors, both alone and in combination with chemotherapy or targeted therapies. Previously, to study safety and tolerability of CBT, Rustin et al. 106 conducted a phase Ib trial that enrolled and treated 46 advanced cancer patients with CBT (10‐min infusion, at doses ranging from 36 to 60 mg/m^2^), 20 h before paclitaxel, carboplatin, or paclitaxel, followed by carboplatin. Grade 3 or 4 neutropenia, thrombocytopenia, grade 1–3 hypertension, and grade 1–3 pain were the typically observed toxicities. Response rates were seen in 22% of patients with OC, esophageal cancer, small‐cell lung cancer, and melanoma. This dose‐escalation trial found this regimen containing CBT well tolerated with adequate premedication supply 106. Combined with radiation, CBT was found to be well tolerated in another phase Ib trial enrolling 39 patients with non‐small‐cell lung cancer (NSCLC), prostate cancer, and squamous cell carcinoma of the head and neck 107. Later, to assess the safety and pharmacokinetics of intravenous CBT as a single dose, He et al. conducted an open‐label, nonrandomized, and dose‐escalation (5, 10, 20, 33, 50, 65, and 85 mg/m^2^ over 30 min) phase I trial enrolling 25 patients with refractory solid tumors. Doses of ≤65 mg/m^2^ given as 30‐min infusions were found to be the maximum tolerated dose in this cohort of East Asian patients 108. Moreover, adding CBT to carboplatin and paclitaxel (63 mg/m^2^ minimum 18 h before carboplatin–paclitaxel repeated q3 wk) was found to be well tolerated in a phase II trial enrolling 44 patients with relapsed OC and appears to have a higher response rate than chemotherapy alone 109. Another phase I trial found that the maximum tolerated dose of CBT is 8.5 mg/m^2^ 110. A tumor response was seen at 14 mg/m^2^, and the recommended dose for phase II trials is 11–14 mg/m^2^ 110. Based on the fact that CBT decreases the vasculature of the surviving tumor clones and, therefore, significantly increases the anticancer activity, another phase I trial using CBT (at 45, 54, or 63 mg/m^2^ on days 1 and 8 and then every 14 days) in combination with bevacizumab (a potent antiangiogenic monoclonal antibody, given at 10 mg/kg on day 8 and at subsequent cycles 4 h after CBT) was conducted by Nathan et al. 111 for patients with advanced melanoma, OC, BC, colorectal adenocarcinoma, and other solid malignancies. Functional imaging with dynamic contrast‐enhanced MRI (DCE‐MRI) showed significantly reduced tumor perfusion and vessel permeability, and this activity was maintained by adding bevacizumab. CBT combined with bevacizumab appears to have a safe and well‐tolerated profile, and 63 mg/m^2^ was recommended for phase II trials 111. Recently, using a dose‐escalation protocol, Liu et al. also investigated the safety and tolerability of CBT in advanced cancer patients with solid tumors. CBT administration was associated with changes in heart rate and blood pressure, and only limited abnormalities in the laboratory tests were seen 112. The maximum tolerated dose was 65 mg/m^2^ 112. Finally, in a randomized phase II trial, Garon et al. 113 showed that CBT in combination with bevacizumab, carboplatin, and paclitaxel appears to have an acceptable toxicity profile in advanced patients with NSCLC.

Unfortunately, these several clinical trials did not demonstrate a sufficient clinical benefit to support its use as a therapeutic alternative. However, these encouraging results on the safety profile of CBT have pushed further investigations in phase II/III trials. Until now, there are three phase III trials conducted by Mateon Therapeutics^®^, Inc. [FACT, FACT2, and Focus trials—NCT00507429, NCT01701349, and NCT02641639, respectively] and still recruiting or withdrawn prior to enrollment.

### Ombrabulin (AVE8062)

**Figure 3:**
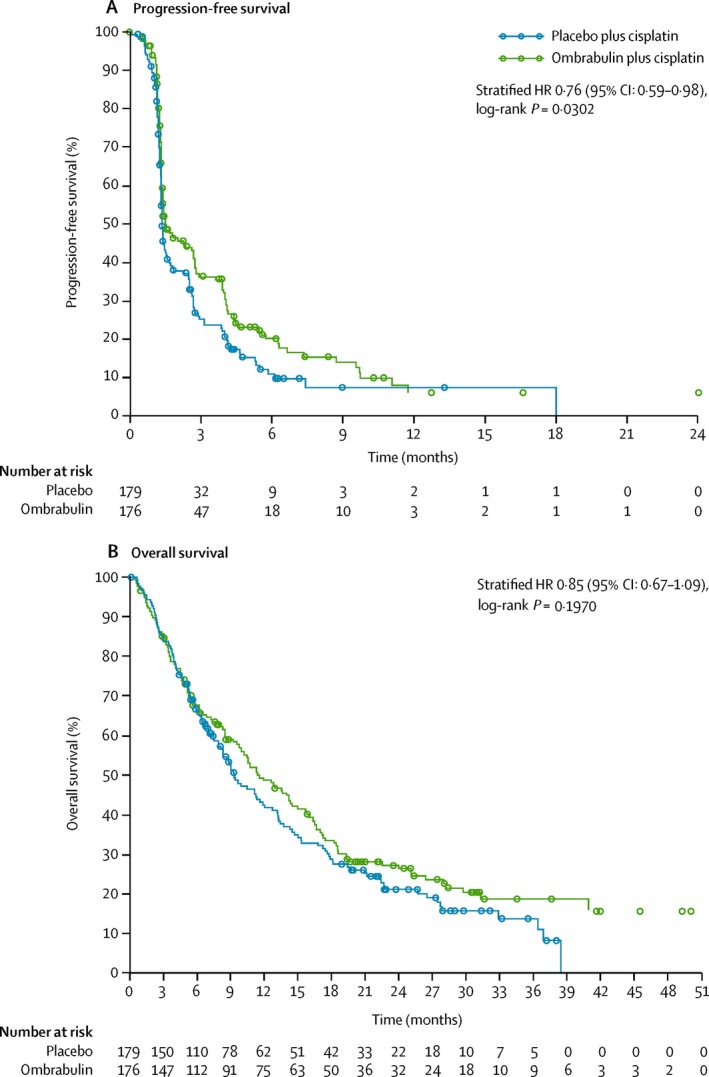
Progression‐free survival (A) and overall survival (B) analyzed in the intention‐to‐treat population. Circles represent where individuals have been censored. HR = hazard ratio. Reprinted from *Lancet Oncol*, 16, Blay et al. Ombrabulin plus cisplatin versus placebo plus cisplatin in patients with advanced soft‐tissue sarcomas after failure of anthracycline and ifosfamide chemotherapy: a randomised, double‐blind, placebo‐controlled, phase 3 trial, 531–40, Copyright (2015), with permission from Elsevier.

Ombrabulin is a soluble analogue of CBT and acts with a similar mechanism of action 36, 114, 115. In preclinical studies, ombrabulin strongly inhibited tumor growth and reduced tumor blood flow by destabilizing tumor vasculature, and potentiated synergistically the action of chemotherapy drugs and radiation 116, 117, 118, 119. Ombrabulin was evaluated in four phase I trials and two randomized phase II/III trials by Sanofi® [NCT01263886 (DISRUPT) and NCT00699517, respectively]. The first one evaluated ombrabulin in combination with paclitaxel–cisplatin in the treatment of advanced STS, and the second evaluated ombrabulin in combination with cisplatin in patients with metastatic NSCLC. Additional trials for other advanced cancers are still ongoing (see Table S3). Ombrabulin (30‐min infusion) was evaluated in a phase I dose‐escalation study (from 6 to 60 mg/m^2^) which enrolled 105 patients with advanced solid tumors 120. At 60 mg/m^2^, the most common adverse events were considered manageable and included grade 3 abdominal pain (at 50 mg/m^2^), grade 3 tumor pain, and grade 3 hypertension. The recommended dose for phase II studies was 50 mg/m^2^. Importantly, the levels of circulating ECs, VEGF (vascular endothelial growth factor), and MMP‐9 (matrix metalloproteinase 9) were found to be significantly increased 6–10 h after intravenous infusion, making them potential biomarkers for investigating ombrabulin efficacy 120. Similarly, a Japanese phase I trial enrolled 15 patients with advanced solid tumors and used q3 wk 30‐min infusion of single‐agent ombrabulin up to 50 mg/m^2^ (15.5, 25, 35, or 50 mg/m^2^) 121. In this trial, ombrabulin was well tolerated with limited cardiovascular side effects. Furthermore, in three additional phase I trials, ombrabulin has recently shown manageable and comparable overlapping toxicities to those observed in previous safety/pharmacokinetic studies 122, 123, 124. Von Pawel et al. 125 randomized 176 patients with metastatic NSCLC in a phase II trial (DISRUPT) to receive ombrabulin (*n* = 88, 35 mg/m^2^) or placebo (*n* = 88) followed by taxane–platinum doublet q3 wk as first‐line therapy. Adding ombrabulin to standard treatment did not show improved PFS when compared to placebo (5.65 vs 5.45 months). Moreover, the two groups had similar OS—11.0 months 125. In a multinational, double‐blind, placebo‐controlled phase III trial, Blay et al. randomized 355 patients with metastatic STS previously treated with anthracycline and ifosfamide. One hundred and seventy‐six patients were randomized to ombrabulin plus cisplatin and 179 patients to placebo plus cisplatin 126. Combining ombrabulin with cisplatin significantly improved PFS (median 1.54 months vs. 1.41 months) but did not demonstrate a sufficient clinical benefit, and no improvement in OS was seen (see Fig. 3 for survival curves) 126. Unfortunately, these disappointing results have forced the discontinuation of developing ombrabulin and its future remains uncertain. Of note, this pivotal study was the only phase III trial conducted on ombrabulin.

### Bryostatin‐1

Bryostatin‐1 is a highly oxygenated macrocyclic lactone isolated from the marine invertebrate *Bugula neritina* 127. Bryostatin‐1 is a potent inhibitor of mammalian target of rapamycin (mTOR) and competitor of protein kinase C (PKC) regulatory domain, a downstream effector of mTOR that plays a key role in cancer cell metabolism, autophagy, cell cycle progression, and angiogenesis as well 128, 129, 130. Importantly, bryostatin‐1 exhibited interesting in vitro and in vivo anticancer activities alone or combined with other antiproliferative agents 127, 131. Currently, there are 41 safety/dose‐finding studies conducted by the National Cancer Institute and registered in the U.S. National Institutes of Health database for HIV, Alzheimer, and various cancers including melanoma, pancreatic cancer, kidney cancer, lymphoma, and ovarian and other gynecological cancers (Table S4). Previous phase I/II trials tested bryostatin‐1 as monotherapy or associated with cytotoxic drugs on a large spectrum of both solid cancers and hematologic malignancies. Results showed that bryostatin‐1 was associated with serious side events including hyponatremia, thrombocytopenia, myalgias, arthralgia, local phlebitis, and fatigue. Also, these trials failed to show clinical benefit with bryostatin‐1 therapy which precludes further phase III trials 132, 133, 134, 135, 136, 137, 138, 139, 140. Recently, combination of weekly bryostatin‐1 (25 μg/m^2^) and paclitaxel (90 mg/m^2^) has shown consistent results with other previous studies 141. In this phase II trial, there were no confirmed responses among 19 patients with locally advanced pancreatic cancer and the response rate did not exceed 30% 141. In another recent phase II trial, combination of bryostatin‐1 (45 μg/m^2^, 72‐h continuous infusion) and cisplatin (50 mg/m^2^) q3 wk was found moderately active (median PFS and OS were 3 and 8.2 months, respectively) in patients with recurrent or persistent OC 142. Fortunately, a recent phase I study of bryostatin‐1 (20 μg/m^2^ q4 wk) and temsirolimus (an approved inhibitor mTOR for the treatment of metastatic renal cell carcinoma) showed optimistic results of PKC inhibition as well as mTOR signaling blockade by acting on both p70‐S6 serine/threonine kinase (mTOR complex 1) and mTOR complex 2 in patients with advanced STS and metastatic renal cell carcinoma 143. It seems that developing selective inhibitors of PKC by modifying the existing bryostatin‐1 using structure–activity relationship studies may potentially enhance the biological activity of temsirolimus (for details, see reviews by Mochly‐Rosen et al. 144, Martin‐Liberal et al. 145, and Garg et al. 146).

Most of these phase I‐II trials were canceled because of serious toxicities (especially myalgias) and because development of bryostatin‐1 is no longer available for clinical investigation. However, bryostatin‐1 appears to have a promising future in HIV/AIDS antiviral “shock and kill” therapy 147.

### Plinabulin (NPI‐2358)

Plinabulin is an antimicrotubule agent and belongs to the halimide class, which is derived from a marine fungus (*Aspergillus ustus*) 148. Mechanistically, it induces loss of cytoskeletal dynamics, structure, and cell–cell cohesion in immature ECs and causes a selective disruption of tumor vascular with a similar mechanism of action to that of CBT 149. Preclinical investigations have shown that NPI‐2358 causes direct cytotoxicity to rapidly proliferating ECs and/or cancer cells by inducing apoptosis through cleavage of poly(ADP‐ribose) polymerase (PARP), activation of caspase‐3, caspase‐8, and caspase‐9, and triggering phosphorylation of the stress response protein JNK (c‐Jun N‐terminal kinase) 150. Only two phase I trials have published data for using plinabulin in advanced NSCLC and lymphoma 151, 152. Mita et al. initiated the first phase I clinical trial (Nereus Pharmaceuticals^®^, Inc., NCT00322608) to assess the safety/pharmacokinetics and to determine the recommended dose of plinabulin for phase II trial in patients with advanced cancers. A favorable safety profile including nausea, vomiting, fever, fatigue, pain, and elevation of transient blood pressure was seen in the majority of the 38 enrolled patients. Interestingly, using DCE‐MRI, this team showed a significant decrease in tumor blood flow (K^Trans^) at 30 mg/m^2^ 151. More details about K^Trans^ and other biomarkers for investigating therapy response to antiangiogenic drugs can be found in two recent reviews by El Bairi et al. 153 and O'Connor et al. 154. The 30 mg/m^2^ dose was selected for further phase II trial in combination with standard chemotherapy. Recently, these data were confirmed by another phase I/II trial (Nereus Pharmaceuticals, Inc., NCT00630110) using a “3 + 3” dose‐escalation design 152. Thirteen patients with advanced NSCLC that had progressed after first‐line chemotherapy were enrolled. The pharmacokinetic evaluation did not show any drug–drug interactions, and 30 mg/m^2^ of plinabulin with 75 mg/m^2^ of docetaxel was recommended for future studies 152. Other phases II/III clinical studies have recently started and are in the process of recruiting patients for lymphoma and lung cancer (Table S5).

### Plitidepsin (Aplidin^®^)

**Figure 4:**
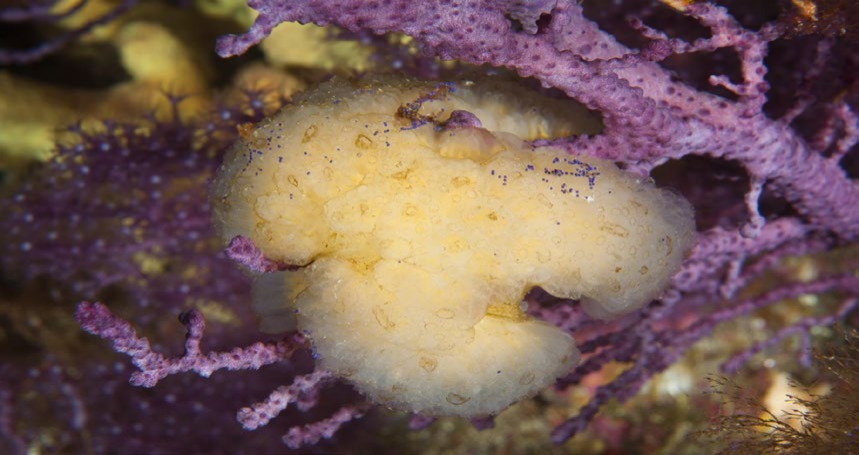
The Mediterranean tunicate *Aplidium albicans* (reused with permission from PharmaMar^®^, S.A., Madrid, Spain).

Plitidepsin is a marine compound also known as dehydrodidembin B. It is a hydrophobic cyclic depsipeptide (solubilized using Cremophor^®^) isolated from the Mediterranean tunicate *Aplidium albicans* (Fig. 4) and marketed by PharmaMar^®^ under the trade name Aplidin^®^ 155, 156, 157. Plitidepsin showed potent pleiotropic actions against a broad spectrum of cancer cell lines and xenograft animal models 157. Effects of plitidepsin encompass angiogenesis, cell cycle arrest, apoptosis by caspase‐3, p38/MAPK, impairment of the eukaryotic elongation factor 1A2 (eEF1A2), subsequent PARP fragmentation, tumor stroma regulation, and sustained JNK activation as well 155, 158, 159. Importantly, JNK activation was demonstrated to be a useful biomarker for evaluating plitidepsin clinical response in vivo 160. Preclinical studies have shown that plitidepsin inhibits angiogenesis by blocking the secretion of VEGF, MMP‐2, MMP‐9, and FGF‐2 161, 162, 163. In addition, combination of plitidepsin with radiation therapy exhibited a potential bystander effect in in vitro studies 164. Plitidepsin was introduced into clinical investigation since 1995 for patients with advanced malignancies 155 and is currently undergoing eight clinical trials (Table S6).

Plitidepsin was evaluated in eight phase I studies (four for plitidepsin alone and four in combination with other chemotherapeutics and targeted agents) for the treatment of several advanced solid tumors and hematologic malignancies. A dose‐escalation Fibonacci scheme was used to determine the recommended dose for phase II trials and safety profile (Table 3). Overall, the safety profile of plitidepsin was considered manageable and included reversible myalgia with oral carnitine treatment, transaminase elevation, fatigue, vomiting, and nausea. The recommended dose ranged from 1.2 to 5 mg/m^2^ (7 mg/m^2^ + carnitine) 165, 166, 167, 168, 169, 170, 171, 172. In phase II trials (summarized in Table 4), plitidepsin showed disappointing activities on several advanced malignancies including liposarcoma, melanoma, thyroid carcinoma, lung cancer, renal cell carcinoma, and other refractory hematologic cancers. Earlier in 2008, Peschel et al. 181 conducted a multicenter phase II single‐arm study that evaluated plitidepsin efficacy using 3‐h continuous infusion of 5 mg/m^2^ biweekly in 21 patients with pretreated locally advanced or metastatic NSCLC. The lack of significant anticancer activity of this single agent in NSCLC (median PFS and OS were 1.2 months and 4.3 months, respectively) was the main cause to close this trial 181. Later, similarly, weekly single‐agent plitidepsin at a dose of 3.2 mg/m^2^ as second‐lined therapy showed no antitumor activity in treating patients with relapsed small‐cell lung cancer (PFS: 1.3 months; median OS: 4.8 months) 177. The same activity was seen in metastatic transitional cell carcinoma of the bladder with 1.4 months and 2.3 months in terms of PFS and OS, respectively 179, in unresectable advanced medullary thyroid carcinoma 175, and recently in liposarcoma (median PFS: 1.6 months; OS: 9.2 months) 173. As yet, a minor clinical response was seen with biweekly plitidepsin at a dose of 5 mg/m^2^ in advanced melanoma (median PFS: 1.3 months; median OS: 3.5 months) 178. In combination with dacarbazine (800 mg/m^2^) in first‐line therapy for advanced melanoma, plitidepsin at 2.4 mg/m^2^ showed some promising results (response rate: 21.4%; PFS: 3.3 months) and probably deserves more investigation in patients with *BRAF‐*wild*‐*type melanoma as demonstrated by another recent phase II trial 169. When considering hematologic malignancies, plitidepsin 5 mg/m^2^ (in a 3‐h infusion biweekly) given in combination with oral dexamethasone showed enhanced and reproducible activity in 47 patients with relapsed and refractory multiple myeloma 176. Moreover, in a multicenter phase II trial, plitidepsin showed encouraging results in noncutaneous peripheral T‐cell lymphoma (an aggressive non‐Hodgkin's lymphoma) 174. This single‐arm trial used plitidepsin at a dose of 3.2 mg/m^2^ as a 1‐h infusion weekly on days 1, 8, and 15 q4 wk. The ORR reached 20.7% including four partial responses and two complete responses 174. Because of these findings in hematologic cancers, plitidepsin (Aplidin^®^) has been granted an orphan drug designation by the Swiss Agency for Therapeutic Products, the EMA, and the FDA for the treatment of patients with multiple myeloma 182. In a recent randomized phase III trial (ADMYRE, NCT01102426) that enrolled 255 heavily pretreated patients with relapsed/relapsed and refractory multiple myeloma, plitidepsin (intravenous 5 mg/m^2^) in combination with dexamethasone (40 mg per os) was compared to dexamethasone alone (40 mg per os). Interestingly, adding plitidepsin was found to significantly reduce the risk of progression or death by 35% 183. Additional randomized studies combining plitidepsin with other anticancer drugs as well as data on predictive biomarkers are awaited.

**Table 3:** Published primary endpoints for phase I trials conducted on plitidepsin

Author/Year	Cancer type	Regimen and administration		Side Events	Recommended dose for phase II studies
Aspeslagh et al. (2017) 165	Refractory solid tumors or lymphomas (*n* = 44)	Plitidepsin + sorafenib (or gemcitabine)	Arm 1: sorafenib/plitidepsin Intravenous plitidepsin at 1.8 mg/m^2^, day 1, day 8, day 15, and, and every 4 weeks (q4 wk) (+ oral administration of sorafenib at two dose levels: day 1: 200 mg twice daily and day 2: 400 mg twice daily) Arm 2: gemcitabine/plitidepsin Intravenous infusion of plitidepsin (day 1–day 2: 1.8 mg/m^2^; day 3: 2.4 mg/m^2^; day 4: 3 mg/m^2^) (+ gemcitabine: day 1: 750 mg/m^2^, day 2–day 4:1000 mg/m^2^). Both agents were administered on day 1, day 8, day 15, and q4 wk	Grade 4 thrombocytopenia Palmar–plantar erythrodysesthesia and erythema Nausea, vomiting, and fatigue Grade 2 transaminase increase	2.4 mg/m^2^ (for combination with gemcitabine) Plitidepsin dose for combination with sorafenib was not defined because of a sponsor decision
Aspeslagh et al. (2016) 166	Refractory solid tumors (*n* = 13)	Plitidepsin + bevacizumab	Intravenous administration of plitidepsin at three dose levels (2.8 mg/m, *n* = 3; 3.8 mg/m, *n* = 4; and 4.8 mg/m, *n* = 6) with bevacizumab (10 mg/kg) at days 1 and 15 of a 28‐day cycle	Grade 3 fatigue Grade 3 myalgia Two grade 2/3 transaminase increases Nausea, vomiting, fatigue, epistaxis, and headache	3.8 mg/m^2^
Salazar et al. (2011) 167	Lymphomas and advanced solid tumors (*n* = 20)	Plitidepsin + carboplatin	Weekly plitidepsin (1‐h intravenous infusion, days 1, 8, and 15; dose escalation starting from 1.8 mg/m^2^) + carboplatin (1‐h intravenous infusion, day 1, after plitidepsin)	Grade 3 transaminase increase Fatigue, myalgia and nausea	2.4 mg/m^2^
Geoerger et al. (2012) 168	Children with refractory or relapsed solid tumors (*n* = 41)	Plitidepsin alone	3‐h intravenous infusion of plitidepsin every 2 weeks (one cycle), standard “3 + 3” design with dose escalation starting from 4, 5 and 6 mg/m^2^	Myalgia, nausea, and vomiting Elevated creatine phosphokinase and transaminase	5 mg/m^2^
Plummer et al. (2013) 169	Advanced melanoma (*n* = 28)	Plitidepsin + dacarbazine	1‐h intravenous infusion of plitidepsin on days 1, 8, and 15, followed by dacarbazine as a 1‐h intravenous infusion on day 1, in cycles of 4 weeks	Grade 4 neutropenic sepsis Grade 3 (fatigue, vomiting, diarrhea, hypersensitivity, respiratory tract infection, weakness, transaminase increase) Grade 4 pancytopenia	2.4 mg/m^2^
Faivre et al. (2005) 170	Advanced malignancies (*n* = 67)	Plitidepsin alone	24‐h intravenous 0.2 mg/m^2^ of plitidepsin every 2 weeks was selected as the starting dose; a modified Fibonacci scheme was used for dose escalation	Grade 2–3 creatine phosphokinase elevation Grade 1–2 myalgia and muscle weakness (at 6 mg/m^2^) Grade 3 myotoxicity (at 5 mg/m^2^, reversible with oral carnitine) Nausea, vomiting, diarrhea, asthenia, and transaminase increase	5 and 7 mg/m^2^ (+carnitine)
Izquierdo et al. (2008) 171	Metastatic solid tumors or non‐Hodgkin's lymphomas (*n* = 48)	Plitidepsin alone	1‐h intravenous infusion of plitidepsin (starting from 0.4 mg/m^2^) given weekly for 3 consecutive weeks during 4‐week treatment cycle	Mild–moderate myalgia, increased creatine phosphokinase levels Grade 3/4 increases in hepatic enzyme levels Fatigue, vomiting/nausea, anorexia, injection site reaction, and pain	3.2 mg/m^2^
Maroun et al. (2006) 172	Refractory solid tumors (*n* = 37)	Plitidepsin alone	1‐h intravenous infusion of plitidepsin (starting dose of 0.08 mg/m^2^) given daily for 5 days q3 wk	Nausea, vomiting, and diarrhea Myalgia, fatigue, and skin rash	1.2 mg/m^2^

**Table 4:** Published phase II trials of plitidepsin

Author/year	Regimen and enrollment	Indication	Response rate (RR)	Progression‐free survival (PFS)	Overall survival (OS)
Toulmonde et al. (2015) 173	Plitidepsin alone (5 mg/m^2^ on days 1‐15 and day 28) (*n* = 24)	Advanced dedifferentiated liposarcoma	–	1.6 months (median)	9.2 months (median)
Plummer et al. (2013) 169	Plitidepsin (2.4 mg/m^2^ on days 1, 8, and 15 every 4 weeks (q4 wk)) + dacarbazine (800 mg/m^2^ q4 wk) (*n* = 38)	Advanced melanoma	21.4%	3.3 months (median)	–
Ribrag et al. (2013) 174	Plitidepsin (1‐h intravenous infusion of 3.2 mg/m^2^ administered weekly on days 1, 8, and 15 q4 wk (*n* = 67)	Relapsed/refractory non‐Hodgkin's lymphoma	20.7%	1.6 months (median)	10.2 months (median)
Baudin et al. (2010) 175	Plitidepsin alone (5 mg/m^2^ as a 3‐h intravenous infusion every 2 weeks (q2 wk)) (*n* = 16)	Unresectable advanced medullary thyroid carcinoma	–	5.3 months (median) (time to disease progression)	Not reached
Mateos et al. (2010) 176	Plitidepsin (5 mg/m^2^ as a 3‐h intravenous infusion q2 wk, and 19 of them added dexamethasone 20 mg/day on days 1–4) (*n* = 51)	Relapsed and refractory multiple myeloma	13% (plitidepsin) and 22% (plitidepsin + dexamethasone)	2.3 months (plitidepsin) and 3.8 months (plitidepsin + dexamethasone)	16.7 months (median, for plitidepsin) and not reached for the second arm
Eisen et al. (2009a) 177	Plitidepsin (1‐h weekly intravenous infusion of 3.2 mg/m^2^) (*n* = 20)	Relapsed small‐cell lung cancer	–	1.3 months	4.8 months (median)
Eisen et al. (2009b) 178	Plitidepsin (3 h of continuous intravenous infusion of 5 mg/m^2^ q2 wk (*n* = 37)	Advanced and relapsed or progressed malignant melanoma	–	1.3 months (median)	3.5 months (median)
Dumez et al. (2009) 179	Plitidepsin (3‐h continuous intravenous infusion of 5 mg/m^2^ q2 wk) (*n* = 21)	Locally advanced or metastatic transitional cell carcinoma of the urothelium	–	1.4 months (median)	2.3 months (median)
Schöffski et al. (2009) 180	24‐h intravenous infusion q2 wk. Arm A (5 mg/m^2^, without L‐carnitine) Arm B (7 mg/m^2^ with L‐carnitine) (*n* = 38)	Unresectable advanced renal cell carcinoma	–	2.1 months (both arms)	7.0 months (arm A) and 7.6 months (arm B)
Peschel et al. (2008) 181	3‐h continuous intravenous infusion of plitidepsin at a dose of 5 mg/m^2^, q2 wk (*n* = 21)	Locally advanced or metastatic non‐small‐cell lung cancer	–	1.2 months (median)	4.3 months (median)

## Mechanisms of Drug Resistance

**Figure 5:**
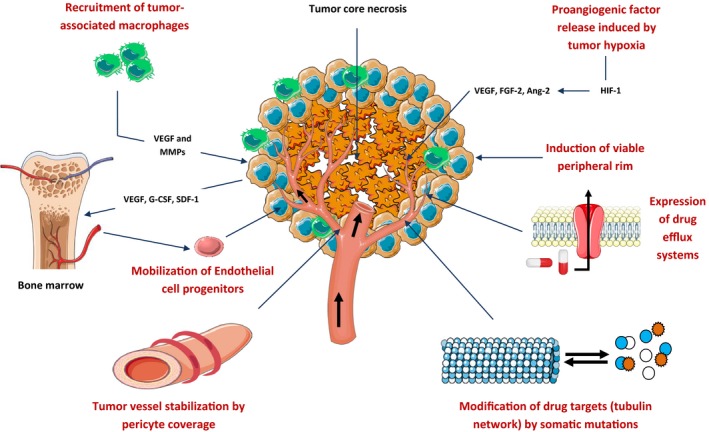
Mechanisms of drug resistance to antivascular‐targeted therapy. For details, see text. Ang‐2, angiopoietin‐2; FGF‐2, fibroblast growth factor 2; G‐CSF, granulocyte colony‐stimulating factor; HIF‐1, hypoxia‐inducible factor 1; MMPs, matrix metalloproteinases; SDF‐1, stromal cell‐derived factor‐1; VEGF, vascular endothelial growth factor.

Despite promising clinical benefits of tumor vessel targeting by these bioactive compounds, drug resistance is inevitable and more invasive tumor regrowth was seen in patients treated with these agents 184, 185. This problem needs to be resolved by deep understanding of various mechanisms of resistance which are currently considered as potential targets for combinatorial approaches (Fig. 5) 184, 186, 187. In addition to the already‐known drug efflux mechanism and somatic mutations in drug targets, it was noted from previous clinical trials, as well as in vivo studies, that cancer cells survive in the periphery of tumors after a single dose of disrupting agent's therapy (the viable rim concept), ensuring the regrowth of the tumor after treatment 184, 188. This type of resistance was explained by the fact that these cells are able to receive oxygen and nutrients to supply their metabolic needs from the surrounding normal tissues. Vascular disrupting agents such as those mentioned earlier lead to the vascular shutdown in the center of the tumors. However, the peripheral vessels remain intact because of the differences observed in their architecture in these two regions. Therefore, combining vascular disrupting agents with other antiangiogenics such as monoclonal antibodies which act on the peripheral blood vessels (bevacizumab, for example) might be promising 184. In this perspective, two clinical trials 111, 189 used fosbretabulin with bevacizumab and found deep vascular damages maintained by this combined regimen. However, these encouraging results were limited by the increased risk of developing hypertension and more associations are to be further investigated. In addition, acute mobilization of bone marrow‐derived progenitors of endothelial cells after treatment by vascular‐targeted agents mediated by chemotactic proteins such as VEGF, G‐CSF, and SDF‐1 (stromal cell‐derived factor‐1, also known as CXCL‐12) was also noted as a driving mechanism of resistance 184, 190, 191, 192. After differentiation, these endothelial cells ensure the restoration of tumor revascularization which explains a rapid regrowth and metastases after treatments. Importantly, pharmacologic targeting of these previous chemokines enhanced the antivascular efficacy and reduced the tumor blood flow and the viable rim size 191. Cancer cell survival and resistance are also dependent on tumor hypoxia caused by vascular shutdown 184, 193. Consequently, tumor cells including the viable rim reprogram their metabolism into high glucose uptake and glycolysis to generate their rapid energetic needs for proliferation and invasion by acquisition of epithelial‐to‐mesenchymal transition phenotype, a concept known as “Warburg effect” (for reviews, see Liberti et al. 194, Muz et al. 195, and Chen et al. 196). Under hypoxic conditions, tumors cells activate HIF‐1 (hypoxia‐inducible factor 1), which in turn acts on many angiogenic pathways including VEGF and other redundant pathways (predominantly FGF‐2 and angiopoietin‐2 (Ang‐2)) 184. Currently, various clinical trials investigating potent HIF‐1 inhibitors are ongoing 197. Recently, TAMs were also suggested as drivers of resistance to vascular targeting as well as poor prognosis 198, 199, 200. Tumor‐infiltrating macrophages are highly proangiogenic and were found to be associated with the recovery of tumor vessels after therapeutic vascular shutdown 201, 202. Recruitment of TAMs promotes fast repair of vascular damages and necrosis via the secretion of VEGF, MMPs, and other cytokines 184. They also increase the mobility of cancer cells and their metastatic ability which explain the observed rapid occurrence of metastases after antivascular therapy and therefore a possible target for drug discovery 184, 203. More recently, another new mechanism was discussed as a potential targetable driver of resistance based on the pericyte coverage of tumor vessels 204, 205. In this perspective, Chen et al. 204 used a prodrug approach to selectively target the tumor pericytes of the peripheral blood vessels via abrogation of their cytoskeleton and observed complete regression of tumors as a surrogate therapy to overcome resistance to these agents. However, along these lines and in light of this growing body of evidence, it seems that cancer cells adapt to any developed therapy in “real‐time” conditions which needs more smart combined therapies.

## Conclusion and Perspectives

Antivascular‐targeted therapeutic modalities using biologically active natural compounds are currently under investigation in various randomized clinical trials. Results from these studies might expand the use of these agents, particularly in combination with other potent anticancer compounds. Because of serious toxicities and lack of efficacy, clinical investigation of bryostatin‐1 and ombrabulin is discontinued. Notably, among dozens of antivascular natural compounds registered in the NIH database, marine‐derived trabectedin (Yondelis^®^) successfully arrived into the market for some advanced sarcomas and platinum‐sensitive recurrent OC. Results from clinical trials investigating plitidepsin, plinabulin, and combretastatin derivatives are still ongoing. Promisingly, new naturally developed antivascular molecules and their derivatives, especially tubulin‐binding agents, have provided potential perspectives in in vitro, in vivo, and early‐phase clinical studies (summarized in Table S7) and may be used in the future in developing novel anticancer drugs. In order to better understand the different effects of these compounds and the physiological angiogenesis effects on tumor development, a crucial in‐depth study is needed. Much of the investigative work has so far been done in cancer chemoprevention, but there is still much more to be done in the use of new approaches, namely the use of natural compounds as starvation tactics against advanced cancers (additional information and useful data can be found in Boxes 1, 2, 3 and Boxes S1 and S2). The following points may guide the future development of these promising molecules: (1) Vascular disruption by natural compounds is an attractive strategy for cancer drug discovery; (2) in the era of evidence‐based medicine, randomized and controlled trials must be the design of all future clinical trials using this strategy to improve their quality; (3) clearly, it is necessary to reduce toxicities associated with these agents as well as to identify predictive biomarkers that will allow investigators to select patients who are most likely to benefit from this treatment; and finally, (4) deep understanding of molecular mechanisms of vascular‐targeted resistance and characterization of the organotypic vasculature of each cancer at the molecular and cellular levels is necessary.

Box 1 **Recommended articles and books of particular interest**

**Table:** 

Review articles and perspectives	DOI
Hollebecque A, Massard C, Soria JC. Vascular disrupting agents: a delicate balance between efficacy and side effects. *Curr Opin Oncol*. 2012;24(3):305–315.	https://doi.org/10.1097/CCO.0b013e32835249de
Ribatti D. Novel angiogenesis inhibitors: addressing the issue of redundancy in the angiogenic signaling pathway. *Cancer Treat Rev*. 2011;37:344‐352.	https://doi.org/10.1016/j.ctrv.2011.02.002
Hoff PM, Machado KK. Role of angiogenesis in the pathogenesis of cancer. *Cancer Treat Rev*. 2012;38(7), 825–833.	https://doi.org/10.1016/j.ctrv.2012.04.006
Rodríguez‐Antona C, Taron M. Pharmacogenomic biomarkers for personalized cancer treatment. *J Intern Med*. 2015;277(2):201–217.	https://doi.org/10.1111/joim.12321
Albini A, Tosetti F, Li VW, et al. Cancer prevention by targeting angiogenesis. *Nat Rev Clin Oncol*. 2012;9(9):498–509.	https://doi.org/10.1038/nrclinonc.2012.120
Atanasov AG, Waltenberger B, Pferschy‐Wenzig EM, et al. Discovery and resupply of pharmacologically active plant‐derived natural products: A review. *Biotechnol Adv*. 2015;33(8):1582–1614.	https://doi.org/10.1016/j.biotechadv.2015.08.001
Nalejska E, Ma¸czynĆska E, Lewandowska MA. Prognostic and Predictive Biomarkers: Tools in Personalized Oncology. *Mol Diagn Ther*. 2014;18(3):273–284.	https://doi.org/10.1007/s40291-013-0077-9
Pritzker KP. Predictive and prognostic cancer biomarkers revisited. *Expert Rev Mol Diagn*. 2015;15(8):971–974.	https://doi.org/10.1586/14737159.2015.1063421
Mita MM, Sargsyan L, Mita AC, et al. Vascular‐disrupting agents in oncology. *Expert Opin Investig Drugs*. 2013;22:317–328.	https://doi.org/10.1517/13543784.2013.759557
Renfro LA, An MW, Mandrekar SJ. Precision oncology: A new era of cancer clinical trials. *Cancer Lett*. 2017;387:121–126.	https://doi.org/10.1016/j.canlet.2016.03.015
Wu G, Wilson G, George J, et al. Overcoming treatment resistance in cancer: current understanding and tactics. *Cancer Lett*. 2017;28;387:69–76.	https://doi.org/10.1016/j.canlet.2016.04.018
Siemann DW. The Unique Characteristics of Tumor Vasculature and Preclinical Evidence for its Selective Disruption by Tumor‐Vascular Disrupting Agents. *Cancer Treat Rev*. 2011;37(1):63–74.	https://doi.org/10.1016/j.ctrv.2010.05.001
Books	
Kim SK. Handbook of Anticancer Drugs from Marine Origin. Springer International Publishing; 2015	https://doi.org/10.1007/978-3-319-07145-9
Saeidnia S. New approaches to natural anticancer drugs. Springer International Publishing; 2015	https://doi.org/10.1007/978-3-319-14027-8
Mehta JL, Mathur P, Dhalla NS (Eds). Biochemical Basis and Therapeutic Implications of Angiogenesis. Springer International Publishing, New York; 2017	https://doi.org/10.1007/978-3-319-61115-0

Box 2 **Useful links and databases**

**Table:** 

Dietmar W. Siemann (PhD) Laboratory (a pioneer in the field of antivascular disrupting therapies)	http://radonc.med.ufl.edu/researchlabs/cancer-biology-laboratories/dietmar-siemann/
The Angiogenesis Foundation	https://angio.org/
Metastasis Research Society	https://www.metastasis-research.org/
The French Angiogenesis Society	http://www.angiogenese.fr/index_en.html
ANGIOGENES: A knowledge database for angiogenesis^a^	http://angiogenes.uni-frankfurt.de/
PubAngioGen database^b^	http://www.megabionet.org/aspd/
Centre for Microvascular Research (William Harvey Research Institute (WHRI))	http://www.whri.qmul.ac.uk/research/inflammation/224-microvascular-research
Tumor angiogenesis and vascular biology laboratory at Mayo Clinic	http://www.mayo.edu/research/labs/tumor-angiogenesis-vascular-biology
Laboratory of Angiogenesis and Vascular Metabolism (VIB Center for Cancer Biology (CCB))	http://www.vib.be/en/research/scientists/Pages/Peter-Carmeliet-Lab.aspx
Angiogenesis and Natural Products Laboratory at the University of Cambridge	https://www.phar.cam.ac.uk/research/fan
Molecular Angiogenesis Laboratory at the University of Liège	http://www.giga.ulg.ac.be/cms/c_22070/en/molecular-angiogenesis-laboratory-home
Ph.D. positions in angiogenesis research	https://www.findaphd.com/search/phd.aspx?keywords=angiogenesis
The International Natural Product Sciences Taskforce	https://www.http://inpst.net
Useful link for Postdoc positions	https://www.findapostdoc.com/
Useful link for research grants and funding	http://www.postgraduatefunding.com/
Open access articles from PubMed Central^®^ related to angiogenesis methods	https://www.ncbi.nlm.nih.gov/pmc/?term=Angiogenesis+methods
Jobs and Ph.D. positions related to angiogenesis research from ResearchGate^®^ website	https://www.researchgate.net/search.Search.html?type=job&query=Angiogenesis
Clinical trials on angiogenesis	https://clinicaltrials.gov/ct2/results?cond=angiogenesis&term=&cntry1=&state1=&recrs

Notes

a Details can be found here: https://www.nature.com/articles/srep32475.

b Details can be found here: https://academic.oup.com/nar/article-lookup/doi/10.1093/nar/gku1139.

Box 3 **Helpful websites**

**Table:** 

National Comprehensive Cancer Network^®^	https://www.nccn.org/
U.S. National Institutes of Health clinical trials database	https://clinicaltrials.gov/
European Society for Medical Oncology (ESMO)	http://esmo.org/
ESMO Clinical Practice Guidelines	http://www.esmo.org/Guidelines
ESMO OncologyPRO^a^	http://oncologypro.esmo.org/
Targeted oncology^®^	http://www.targetedonc.com/
OncLive^®^	http://www.onclive.com/
American Society of Clinical Oncology (ASCO)	https://www.asco.org/
ASCO Clinical Practice Guidelines	https://www.asco.org/practice-guidelines/quality-guidelines/guidelines
Professional networking site of the ASCO	http://connection.asco.org/
The Conquer Cancer Foundation^®^	https://www.conquer.org/
American Cancer Society (ACS)	https://www.cancer.org/
American Association for Cancer Research (AACR)	http://www.aacr.org/Pages/Home.aspx
*e*cancer^b^	http://ecancer.org/
European CanCer Organisation (ECCO)	http://www.ecco-org.eu/
European Association for Cancer Research (EACR)	https://www.eacr.org/

a A scientific and educational tool offering the latest information in new cancer treatments, prevention strategies and research.

b A vast online free knowledge bank to the whole oncology community (available in both English and Spanish).

## Conflict of Interest

None declared.

## Supporting information


**Table S1.** Trabectedin phase III trials.
**Table S2.** Combretastatin clinical trials.
**Table S3.** Ombrabulin clinical trials.
**Table S4.** Completed clinical trials of bryostatin‐1.
**Table S5.** Plinabulin clinical trials.
**Table S6.** Plitidepsin clinical trials.
**Table S7.** Promising anti‐vascular natural compounds and their derivatives tested in (pre)clinical studies for cancer drug discovery.
**Figure S1.** Disposition of trabectedin clinical trials by phase number.
**Figure S2.** Disposition of OVA‐301 phase III study.
**Figure S3.** Mechanism of action of combretastatin.
**Figure S4.** Survival of patients with soft tissue sarcoma according to tumor tissue markers.
**Figure S5.** Survival of ovarian cancer patients treated with trabectedin.
**Box S1.** Useful list of Medline‐indexed and highly accessed journals studying angiogenesis and related oncology clinical trials.
**Box S2.** Additional useful reviews of particular interest.
