# Supplementary material for: Starvation tactics using natural compounds for advanced cancers: pharmacodynamics, clinical efficacy, and predictive biomarkers
Source: Cancer Med. 2018 May 6;7(6):2221–46. doi: 10.1002/cam4.1467 (PMC6010871; doi:10.1002/cam4.1467)
Supplement: Supplementary file 1 — Table S1. Trabectedin phase III trials. Table S2. Combretastatin clinical trials. Table S3. Ombrabulin clinical trials. Table S4. Completed clinical trials of bryostatin‐1. Table S5. Plinabulin clinical trials. Table S6. Plitidepsin clinical trials. Table S7. Promising anti‐vascular natural compounds and their derivatives tested in (pre)clinical studies for cancer drug discovery. Figure S1. Disposition of trabectedin clinical trials by phase number. Figure S2. Disposition of OVA‐301 phase III study. Figure S3. Mechanism of action of combretastatin. Figure S4. Survival of patients with soft tissue sarcoma according to tumor tissue markers. Figure S5. Survival of ovarian cancer patients treated with trabectedin. Box S1. Useful list of Medline‐indexed and highly accessed journals studying angiogenesis and related oncology clinical trials. Box S2. Additional useful reviews of particular interest. [file CAM4-7-2221-s001.docx]

**Supplemental Material for:**

**Starvation Tactics Using Natural Compounds for Advanced Cancers: Pharmacodynamics, Clinical Efficacy and Predictive Biomarkers**

**Supplemental Tables**

**Supplementary Table S1. Trabectedin phase III trials**

| **Indication** | **Interventions** | **Sponsor** |
| --- | --- | --- |
| [Advanced and Relapsed Ovarian Cancer](https://clinicaltrials.gov/ct2/show/NCT00113607?term=Yondelis&no_unk=Y&rank=1) | Trabectedin, doxorubicin, and dexamethasone | Johnson & Johnson Pharmaceutical Research & Development, L.L.C. in collaboration with PharmaMar |
| Advanced or Metastatic Liposarcoma or Leiomyosarcoma | Trabectedin and dacarbazine | Xian-Janssen  Pharmaceutical Ltd |
| [Translocation-Related Sarcomas (TRS)](https://clinicaltrials.gov/ct2/show/NCT00796120?term=trabectedin&no_unk=Y&rank=26) | Trabectedin, doxorubicin, and ifosfamide | Johnson & Johnson Pharmaceutical Research Development, L.L.C. in collaboration with PharmaMar |
| Ovarian, peritoneal  and fallopian  tube cancers | Trabectedin, doxorubicin, and dexamethasone | Janssen Research & Development, LLC;  in collaboration with PharmaMar |
| Advanced Liposarcoma or Leiomyosarcoma | Trabectedin and dacarbazine | Janssen Research & Development, LLC;  in collaboration with PharmaMar |
| [Advanced or Metastatic Soft Tissue Sarcoma](https://clinicaltrials.gov/ct2/show/NCT01189253?term=trabectedin&no_unk=Y&rank=36) | Trabectedin and doxorubicin | European Organisation for Research and Treatment of Cancer (EORTC) in collaboration with Sarcoma Alliance for Research through Collaboration |
| Soft Tissue Sarcoma | Trabectedin and dexamethasone | Gustave Roussy, Cancer Campus, Grand Paris |
| Locally Advanced or Metastatic Soft Tissue Sarcoma | Trabectedin alone | Janssen Research & Development, LLC |
| Ovarian cancer | Trabectedin, carboplatin, and doxorubicin | Mario Negri Institute for Pharmacological Research  in collaboration with PharmaMar and Averion International Corporation |
| Localized high-risk soft tissue sarcomas (STS) | Trabectedin, epirubicin, gemcitabine, ifosfamide, etoposide and gemcitabine | Italian Sarcoma Group in collaboration with Groupe Sarcomes Français (GSF-GETO) and Grupo Espanol de Investigacion en Sarcomas |

Source: NIH database ([www.clinicaltrials.gov](http://www.clinicaltrials.gov))

**Supplementary Table S2. Combretastatin clinical trials**

| **Title of the study** | **Identifier** | **Interventions** | **Phase** | **Status** | **Sponsor** |
| --- | --- | --- | --- | --- | --- |
| Safety and effectiveness of Combretastatin A-4 Phosphate Combined With Chemotherapy in Advanced Solid Tumors | NCT00113438 | Arm 1: Combretastatin A-4 Phosphate (CA4P) (45 mg/m^2^) + paclitaxel and carboplatin  Arm 2: CA4P (60 mg/m^2^) + paclitaxel and carboplatin | II | Completed | Mateon Therapeutics |
| Combretastatin A4 Phosphate in Treating Patients With Advanced Anaplastic Thyroid Cancer | NCT00060242 | CA4P (45 mg/m^2^) | II | Completed | Case Comprehensive  Cancer Center in collaboration with NCI |
| Safety Study of Increasing Doses of Combretastatin in Combination With Bevacizumab (Avastin) in Patients With Advanced Solid Tumors | NCT00395434 | CA4P (dose-escalation schedule) + bevacizumab | I | Completed | Mateon Therapeutics |
| Fosbretabulin or Placebo in Combination With Carboplatin/Paclitaxel in Anaplastic Thyroid Cancer (FACT2) | NCT01701349 | Arm 1: CA4P (60 mg/m^2^)  + paclitaxel + carboplatin  Arm 2: Placebo + paclitaxel + carboplatin | III | Withdrawn prior to enrollment | Mateon Therapeutics |
| Study of Combretastatin and Paclitaxel/Carboplatin in the Treatment of Anaplastic Thyroid Cancer (FACT) | NCT00507429 | Arm 1: CA4P (60 mg/m^2^) + carboplatin + paclitaxel  Arm 2: Carboplatin + paclitaxel | II/III | Terminated  (Low rate of subject accrual) | Mateon Therapeutics |
| Safety Study of Increasing Doses of Combretastatin A1 Diphosphate (OXi4503) as Monotherapy in Subjects With Hepatic Tumor Burden (OXi4503) | NCT00960557 | Combretastatin A1 Diphosphate (OXi4503) (dose escalation schedule) | I | Completed | Mateon Therapeutics |
| A Phase I Clinical Trial of OXi4503 for Relapsed and Refractory AML and MDS | NCT01085656 | OXi4503 (dose escalation schedule) | I | Terminated | University of Florida  in collaboration with The Leukemia and Lymphoma Society |
| A Safety and Efficacy Study of Carboplatin, Paclitaxel, Bevacizumab and CA4P in Non-Small Cell Lung Cancer (FALCON) | NCT00653939 | Arm 1: Carboplatin + paclitaxel + bevacizumab  Arm 2: CA4P (60 mg/m2) + carboplatin + paclitaxel + bevacizumab | II | Completed | Mateon Therapeutics |
| Combretastatin A4 Phosphate in Treating Patients With Advanced Solid Tumors | NCT00003768 | CA4P (dose-escalation schedule) | I | Completed | Case Comprehensive  Cancer Center in collaboration with NCI |
| Chemotherapy in Treating Patients With Solid Tumors | NCT00003698 | CA4P (dose-escalation schedule) | I | Completed | University of Glasgow in collaboration with NCI |
| A Phase 2 Study of Fosbretabulin in Subjects with Pancreatic or Gastrointestinal Neuroendocrine Tumors with Elevated Biomarkers (GI-NETor PNET) | NCT02132468 | CA4P (60 mg/m^2^) | II | Completed | Mateon Therapeutics |
| Rollover Protocol for Subjects Who Have Responded on Study 4218s - A Phase 2 Study | NCT02279602 | CA4P (60 mg/m^2^) | II | Completed | Mateon Therapeutics |
| Fosbretabulin or Placebo in Combination With Carboplatin/Paclitaxel in Anaplastic Thyroid Cancer (FACT2) | NCT01701349 | Arm 1: CA4P (60 mg/m^2^) + paclitaxel + carboplatin  Arm 2: Placebo + paclitaxel + carboplatin | III | Withdrawn prior to enrollment | Mateon Therapeutics |
| FOCUS: PCC + Bevacizumab + CA4P Versus PCC + Bevacizumab + Placebo for Subjects With Platinum Resistant Ovarian Cancer | NCT02641639 | Arm 1: CA4P (60 mg/m^2^) + paclitaxel + pegylated liposomal doxorubicin (PLD) + bevacizumab  Arm 2: Paclitaxel + PLD + bevacizumab + placebo | II/III | Recruiting | Mateon Therapeutics |
| Dose Escalation of OXi4503 as Single Agent and Combination With Cytarabine w/Subsequent Ph 2 Cohorts for AML and MDS (AML) | NCT02576301 | Phase I: OXi4503 (dose-escalation schedule) + cytarabine  Phase 2: OXi4503 + cytarabine (1g/m^2^) | I/II | Recruiting | Mateon Therapeutics |

Source: NIH database ([www.clinicaltrials.gov](http://www.clinicaltrials.gov), accessed 11/09/2017). Titles of the trials were used as shown by the database and doses of different schedules were included in the table when found, when not found, external resources were used.

**Supplementary Table S3. Ombrabulin clinical trials**

| **Title of the Study** | **Identifier** | **Interventions** | **Phase** | **Status** | **Sponsor** |
| --- | --- | --- | --- | --- | --- |
| Dose Escalation, Safety and Pharmacokinetic Study of AVE8062 Combined With Cisplatin in Patients With Solid Tumors | NCT01021150 | Ombrabulin (dose-escalation schedule) + cisplatin (75 mg/m^2^) | I | Completed | Sanofi |
| Dose-escalation, Safety, Pharmacokinetics Study of AVE8062 Combined With Bevacizumab in Patients With Advanced Solid Tumors | NCT01193595 | Ombrabulin (dose-escalation schedule) + bevacizumab (10 mg/kg and 15 mg/kg) | I | Completed | Sanofi |
| Dose Escalation, Safety and Pharmacokinetic Study of AVE8062 Combined With Docetaxel in Patients With Advanced Solid Tumors | NCT01907685 | Ombrabulin (11.5 to 42 mg/m^2^) + docetaxel (75 and 100 mg/m^2^) | I | Completed | Sanofi |
| Study of Ombrabulin in Patients With Platinum-Sensitive Recurrent Ovarian Cancer Treated With Carboplatin/Paclitaxel (OPSALIN) | NCT01332656 | Arm 1: Ombrabulin ( 35 mg/m^2^) + paclitaxel and carboplatin  Arm 2: Placebo, paclitaxel and carboplatin | II | Completed | Sanofi |
| A Dose-escalation Study of Ombrabulin in Combination With Paclitaxel and Carboplatin in Patients With Advanced Solid Tumors | NCT01293630 | Ombrabulin (dose-escalation schedule) + paclitaxel and carboplatin | I | Completed | Sanofi |
| Dose Escalation, Safety and Pharmacokinetic Study of AVE8062 in Patients With Solid Tumors | NCT00968916 | Ombrabulin (dose-escalation schedule:15.5 mg/m^2^, 25 mg/m^2^, 35 mg/m^2^ and 50 mg/m^2^) | I | Completed | Sanofi |
| AVE8062 in Combination With Platinum-taxane Doublet in Advanced Solid Tumor | NCT00719524 | Ombrabulin (dose-escalation schedule) + chemotherapy (cisplatin/carboplatin + docetaxel/paclitaxel) | I | Completed | Sanofi |
| Trial of Ombrabulin (AVE8062) in Combination With Taxane and Platinum in Patients With Non-small Cell Lung Cancer (DISRUPT) | NCT01263886 | Arm 1: Ombrabulin (35 mg/m^2^) + docetaxel + cisplatin or paclitaxel + carboplatin  Arm 2: Placebo + docetaxel + cisplatin or paclitaxel + carboplatin | I | Completed | Sanofi |
| A Study to Investigate the Disposition of Radio-labeled AVE8062 Compound Administered as a 30-minute IV Infusion to Patients With Advanced Solid Tumor | NCT01063946 | [14C]-Ombrabulin (25 mg/m²) + non-radiolabelled ombrabulin + cisplatin | I | Completed | Sanofi |
| Dose Escalation, Safety and Pharmacokinetic Study of AVE8062 Combined With Docetaxel and Cisplatin in Patients With Solid Tumors | NCT01095302 | Ombrabulin (dose-escalation schedule) + docetaxel + cisplatin | I | Completed | Sanofi |
| A Study of AVE8062 in Advanced-stage Soft Tissue Sarcoma After Failure of Anthracycline and Ifosfamide Chemotherapies | NCT00699517 | Arm 1: Ombrabulin (25 mg/m^2^) + cisplatin (75 mg/m^2^)  Arm 2: Placebo + cisplatin (75 mg/m^2^) | III | Completed | Sanofi |

Source: NIH database ([www.clinicaltrials.gov](http://www.clinicaltrials.gov), as of: 10-08-2017)

**Supplementary Table S4. Completed clinical trials of bryostatin-1**

| **Title of the study** | **Phase** | **Interventions** | **Sponsor** |
| --- | --- | --- | --- |
| [Bryostatin 1 plus Vincristine in treating patients With recurrent or refractory HIV-related lymphoma](https://clinicaltrials.gov/ct2/show/NCT00022555?term=Bryostatin-1&no_unk=Y&rank=1) | I | Bryostatin-1 +   vincristine | National Cancer Institute (NCI) |
| [Paclitaxel and Bryostatin 1 in treating patients with advanced pancreatic cancer](https://clinicaltrials.gov/ct2/show/NCT00031694?term=Bryostatin-1&no_unk=Y&rank=2) | II | Bryostatin-1 + paclitaxel | NCI |
| [Temsirolimus and Bryostatin-1 in treating patients With unresectable or metastatic solid tumors](https://clinicaltrials.gov/ct2/show/NCT00112476?term=Bryostatin-1&no_unk=Y&rank=3) | I | Bryostatin-1 + temsirolimus | NCI |
| [Bryostatin-1 plus Vincristine in treating patients with progressive or relapsed non-Hodgkin’s lymphoma after bone marrow or stem cell transplantation](https://clinicaltrials.gov/ct2/show/NCT00058305?term=Bryostatin-1&no_unk=Y&rank=6) | II | Bryostatin-1 +   Vincristine | NCI |
| [Interleukin-2 and Bryostatin 1 in treating patients with advanced kidney cancer](https://clinicaltrials.gov/ct2/show/NCT00032188?term=Bryostatin-1&no_unk=Y&rank=7) | II | [Interleukin-2 + bryostatin-1](https://clinicaltrials.gov/ct2/show/NCT00032188?term=Bryostatin-1&no_unk=Y&rank=7) | NCI |
| [Bryostatin and Vincristine in B-Cell malignancies](https://clinicaltrials.gov/ct2/show/NCT00003166?term=Bryostatin-1&no_unk=Y&rank=8) | I | Bryostatin-1 +   vincristine | NCI |
| [Bryostatin 1 and Cisplatin in treating patients with advanced recurrent or residual ovarian epithelial, fallopian tube, or primary peritoneal cancer](https://clinicaltrials.gov/ct2/show/NCT00006942?term=Bryostatin-1&no_unk=Y&rank=9) | II | Bryostatin-1 + cisplatin | NCI |
| [Bryostatin 1 and Cisplatin in treating patients with metastatic or unresectable stomach cancer](https://clinicaltrials.gov/ct2/show/NCT00006389?term=Bryostatin-1&no_unk=Y&rank=10) | II | Bryostatin-1 + cisplatin | NCI |
| [A study of All-Trans Retinoic Acid (ATRA) and Bryostatin in patients with acute myeloid leukemia (AML) and myelodysplastic syndrome (MDS)](https://clinicaltrials.gov/ct2/show/NCT00136461?term=Bryostatin-1&no_unk=Y&rank=12) | II | Bryostatin-1 + All-trans retinoic  acid | NCI |
| [Chemotherapy Plus Sargramostim in treating patients with refractory myeloid cancer](https://clinicaltrials.gov/ct2/show/NCT00012376?term=Bryostatin-1&no_unk=Y&rank=13) | I | Bryostatin-1 + sargramostim | NCI |
| Bryostatin-1 Plus Gemcitabine in treating patients with advanced cancer | I | Bryostatin-1 + gemcitabine | Barbara Ann Karmanos Cancer Institute in collaboration of NCI |
| Bryostatin-1  in treating patients with metastatic kidney cancer | II | Bryostatin-1 alone | NCI |
| Bryostatin-1 in treating patients with recurrent Non-Hodgkin's lymphoma | II | Bryostatin-1 + single-agent chemotherapy | NCI |
| Bryostatin-1 plus Paclitaxel in treating patients with stage IIIB, stage IV, or recurrent Non-small cell lung cancer | II | Bryostatin-1 + paclitaxel | University of Chicago in collaboration with NCI |
| Paclitaxel and Bryostatin-1 in treating patients with metastatic prostate cancer | II | Bryostatin-1 + paclitaxel | NCI |
| Bryostatin 1 and Rituximab in treating Patients with B-Cell Non-Hodgkin's lymphoma or chronic lymphocytic leukemia | II | Bryostatin-1 + rituximab | NCI |
| Bryostatin-1  in treating patients with progressive kidney cancer | II | Bryostatin-1 alone | Cancer Research UK in collaboration with NCI |
| Interleukin-2 plus Bryostatin-1  in treating patients with melanoma or kidney cancer | I | Interleukin-2 + bryostatin-1 | Virginia Commonwealth University in collaboration with NCI |
| Bryostatin-1 in treating patients with stage IV breast cancer | II | Bryostatin-1 alone | University of Colorado, Denver in collaboration with NCI |
| Bryostatin-1  in treating patients with metastatic colorectal cancer | II | Bryostatin-1 alone | Barbara Ann Karmanos Cancer Institute in collaboration with NCI |
| Bryostatin-1  in treating patients with myelodysplastic syndrome | II | Bryostatin-1 alone | Barbara Ann Karmanos Cancer Institute in collaboration with NCI |
| Bryostatin-1 in treating patients with relapsed multiple myeloma | II | Bryostatin-1 | Barbara Ann Karmanos Cancer Institute in collaboration with NCI |
| Bryostatin-1  in treating patients with recurrent or refractory Hodgkin's disease | II | Bryostatin-1 alone | Barbara Ann Karmanos Cancer Institute in collaboration with NCI |
| Bryostatin-1 in treating patients with ovarian epithelial cancer | II | Bryostatin-1 | University of Glasgow  in collaboration with NCI |
| Bryostatin-1 plus cisplatin in treating patients with metastatic or unresectable Cancer | I | Bryostatin-1 + cisplatin | New York University School of Medicine in collaboration with NCI |
| Bryostatin-1  in treating patients with metastatic or recurrent head and neck cancer | II | Bryostatin-1 alone | Memorial Sloan Kettering Cancer Center in collaboration with NCI |
| Bryostatin-1 plus Cladribine in treating patients with relapsed chronic lymphocytic leukemia | I | Bryostatin-1 + cladribine | Barbara Ann Karmanos Cancer Institute in collaboration with NCI |
| Bryostatin-1 plus Paclitaxel in treating patients with locally advanced or metastatic esophageal cancer or stomach cancer | II | Bryostatin-1 + paclitaxel | Memorial Sloan Kettering Cancer Center in collaboration with NCI |
| Bryostatin-1 and Cytarabine in treating patients with relapsed acute myelogenous leukemia | II | Bryostatin-1 + cytarabine | Virginia Commonwealth University in collaboration with NCI |
| Bryostatin-1 in treating patients with relapsed Non-Hodgkin's lymphoma or chronic lymphocytic leukemia | II | Bryostatin-1 + vincristine | Barbara Ann Karmanos Cancer Institute in collaboration with NCI |
| Bryostatin-1 and high dose Cytarabine in treating patients with refractory or relapsed leukemia or lymphoma | I | Bryostatin-1 + cytarabine | Virginia Commonwealth University in collaboration with NCI |
| Bryostatin-1 plus Paclitaxel and Cisplatin in treating patients with advanced solid tumors | I | Bryostatin-1 + Paclitaxel + cisplatin | Memorial Sloan Kettering Cancer Center in collaboration with NCI |
| Bryostatin-1  and Interleukin-2 in treating patients with refractory solid tumors or lymphoma | I | Bryostatin-1 + Interleukin-2 | National Institute on Aging (NIA) in collaboration with NCI |
| Combination chemotherapy in treating patients with unresectable locally advanced or metastatic stomach cancer | II | Bryostatin-1 + paclitaxel | M.D. Anderson Cancer Center in collaboration with NCI |
| Cisplatin plus Bryostatin-1 in treating patients with advanced cancer | I | Bryostatin-1 + cisplatin | Lombardi Cancer Research Center in collaboration with NCI |
| Bryostatin + Fludarabine in treating patients with chronic lymphocytic leukemia or relapsed indolent Non-Hodgkin's lymphoma | I | Bryostatin-1 + fludarabine | Virginia Commonwealth University in collaboration with NCI |

Source: NIH database ([www.clinicaltrials.gov](http://www.clinicaltrials.gov))

**Supplementary Table S5. Plinabulin clinical trials**

| **Indication** | **Identifier** | **Phase** | **Interventions** | **Starting year** | **Status** | **Sponsor** |
| --- | --- | --- | --- | --- | --- | --- |
| Nivolumab and Plinabulin in Treating Patients With Stage IIIB-IV, Recurrent, or Metastatic Non-small Cell Lung Cancer | NCT02846792 | I/II | Nivolumab +  plinabulin | 2016 | Recruiting | University of Washington in collaboration with National cancer institute |
| Plinabulin vs. Pegfilgrastim in Reducing the Duration of Severe Neutropenia in Breast Cancer Patients Receiving Myelosuppressive Chemotherapy With Docetaxel, Doxorubicin, and Cyclophosphamide (TAC) | NCT03294577 | II/III | Plinabulin vs. pegfilgrastim | 2017 | Not yet open for participant recruitment | BeyondSpring Pharmaceuticals Inc. |
| Plinabulin vs. Pegfilgrastim in Patients With Solid Tumors Receiving Docetaxel Myelosuppressive Chemotherapy (Protective-1) | NCT03102606 | II/III | Plinabulin  vs. pegfilgrastim | 2017 | Recruiting | BeyondSpring Pharmaceuticals Inc.  in collaboration with  Chiltern International Inc. and ICON plc |
| Phase 1/2 Study of Vascular Disrupting Agent NPI-2358 + Docetaxel in Patients With Advanced Non-Small Cell Lung Cancer | NCT00630110 | I/II | Plinabulin + docetaxel | 2008 | Completed | Nereus Pharmaceuticals, Inc. |
| Assessment of Docetaxel + Plinabulin Compared to Docetaxel + Placebo in Patients With Advanced NSCLC With at Least One Measurable Lung Lesion (DUBLIN-3) | NCT02504489 | III | Docetaxel + plinabulin vs. docetaxel | 2015 | Recruiting | BeyondSpring Pharmaceuticals Inc. |
| Nivolumab in Combination With Plinabulin in Patients With Metastatic Non-Small Cell Lung Cancer (NSCLC) | NCT02812667 | I | Nivolumab +  plinabulin | 2016 | Recruiting | Lyudmila Bazhenova, M.D., in collaboration with BeyondSpring Pharmaceuticals Inc. |
| Study of the Vascular Disrupting Agent NPI-2358 in Patients With Advanced Solid Tumors or Lymphoma | NCT00322608 | I | plinabulin | 2006 | Completed | Nereus Pharmaceuticals, Inc. |

Source: NIH database ([www.clinicaltrials.gov](http://www.clinicaltrials.gov), as of 27-Sept-2017)

**Supplementary Table S6. Plitidepsin clinical trials**

| **Title of the study** | **Identifier** | **Interventions** | **Phase** | **Status** | **Sponsor** |
| --- | --- | --- | --- | --- | --- |
| Multicenter Trial to Treat Patients With Relapsed/Refractory Aggressive Non Hodgkin Lymphoma | NCT00884286 | Intravenous Aplidin^®^ at a starting dose of 3.2 mg/m^2^ (1-hour infusion, on days 1, 8 and 15, every 4 weeks (q4wk)) | II | Completed | PharmaMar |
| A Study of Aplidin (Plitidepsin) in Subjects With Advanced Prostate Cancer | NCT00780975 | Intravenous Aplidin^®^ at a starting dose of 5 mg/m^2^ (3-hours infusion, every 2 weeks (q2wk)) | II | Terminated (poor recruitment) | PharmaMar |
| Study of Plitidepsin (Aplidin®) in Combination With Bortezomib and Dexamethasone in Patients With Multiple Myeloma | NCT02100657 | Intravenous Aplidin^®^ (dose-escalation schedule, 3-hour infusion on day 1 and 15, q4wk) + subcutaneous bortezomib (days 1, 4, 8 and 11, q4wk)+ oral dexamethasone (days, 1, 8, 15 and 22, q4wk) | I |  | PharmaMar |
| Study of Plitidepsin in Combination With Sorafenib or Gemcitabine in Patients With Advanced Solid Tumors or Lymphomas | NCT00788099 | Arm 1: Intravenous Aplidin^®^ (1-hour infusion, on days 1, 8 and 15 (q4wk) + continuous oral sorafenib (twice daily, q4wk)  Arm 2: Intravenous Aplidin^®^ (1-hour infusion, on days 1,8 and 15, q4wk) + gemcitabine | I | Completed | PharmaMar |
| A Study of Aplidin (Plitidepsin) 3 h iv in Subjects With Relapsing or Refractory Multiple Myeloma | NCT00229203 | Intravenous Aplidin^®^ (5 mg/m^2^, 3-hours infusion, q2wk) | II | Completed | PharmaMar |
| Clinical Study of Plitidepsin (Aplidin®) in Combination With Cytarabine in Patients With Relapsed/Refractory Leukemia | NCT00780143 | Intravenous Aplidin^®^ (at starting dose of  0.54 mg/m^2^, 1-hour infusion, every 3 weeks (q3wk)) + cytarabine (1g/m^2^ daily for 5 days) | I/II | Terminated (poor recruitment) | PharmaMar |
| Trial of Plitidepsin (Aplidin®) in Combination With Bortezomib and Dexamethasone in Multiple Myeloma Patients Double Refractory to Bortezomib and Lenalidomide | NCT03117361 | Intravenous Aplidin^®^ (3-hour infusion on days 1 and 15, q4wk) + subcutaneous bortezomib on days 1, 4, 8 and 11, q4wk) + oral dexamethasone on days 1,8,15 and 22, q4wk) | II | Recruiting | PharmaMar |
| Aplidin - Dexamethasone in Relapsed/Refractory Myeloma (ADMYRE) | NCT01102426 | Arm 1: Intravenous Aplidin^®^ (3-hours infusions of 5 mg/m^2^ on days 1 and 15, q4wk) + oral dexamethasone (40 mg on days 1, 8, 15 and 22, q4wk)  Arm 2: Dexamethasone  (40 mg orally on days 1, 8, 15 and 22, q4wk) | III | Ongoing | PharmaMar |

Source: NIH database ([www.clinicaltrials.gov](http://www.clinicaltrials.gov), as of 27-Sept-2017)

**Supplementary Table S7. Promising anti-vascular natural compounds and their derivatives tested in (pre)clinical studies for cancer drug discovery**

| **Anti-vascular natural compounds** | **Source** | **Mechanism of action/indication** | **Used in early clinical trials? ^#^** | **Sponsor** | **References** |
| --- | --- | --- | --- | --- | --- |
| **Epidithiodiketopiperazines** | Fungal secondary metabolites | Disruption of HIF-1α/p300 complex in a preclinical model of prostate cancer | No | - | Reece et al. 2014 |
| **DMXAA (Vadimezan, ASA404)** | Flavonoids | Disruption of tumor vasculature by multikinases inhibition and cytokine induction | Yes^†^ | Novartis | Buchanan et al.2012 (for review, see: Siemann, 2011 and Porcù et al. 2014) |
| **Hemiasterlin** | Marine sponges | Inhibition of tubulin polymerization^k^ | Yes (its analogue E7974)  NCT00165802  NCT00130169  NCT00121732 | Eisai Inc. | Hsu et al. 2012;  Kuznetsov et al. 2009 |
| **Dolastatin 10** (and its analogues) | Sea hare (*Dolabella auricularia*) | Suppression of microtubule dynamics | Yes^ǂ^ | National Cancer Institute (NCI) | Müller et al. 2014; Gajula et al. 2013 (for review, see: Fanale et al. 2015) |
| **Resveratrol** | Grapes | Inhibition of VEGF signaling | Yes  NCT00920803  NCT01476592  NCT00433576 | GlaxoSmithKline | Howells et al. 2011; Trapp et al. 2010 (for review, see: Shanmugam et al. 2017 and Block et al. 2015) |
| **Celastrol** | *Trypterigium wilfordii* | Inhibition of HIF-1α pathway | No | - | Huang et al. 2011 |
| **Withaferin A** | *Withania somnifera* | Knockdown of Notch-2 | No | - | Mohan et al. 2004; Kim et al. 2016 |
| **Lurbinectedin** (a synthetic analogue of trabectedin) | - | Inhibition of the production of inflammatory/growth factors (CCL2, CXCL8 and VEGF) and depletion of tumor associated macrophages | Yes^¥^ | PharmaMar | Elez et al. 2014; Belgiovine et al. 2017; Céspedes et al. 2016 |
| **Curcumin** | *Curcuma longa* | Attenuation of FoxM1 expression and its downstream genes, such as cyclin B1, CDK2, S-phase kinase-associated protein 2, Cdc25B, survivin, Bcl-2, MMP-2, MMP-9, and VEGF | Yes^§^ | - | Zhang et al. 2014 (for review: Wang et al. 2015) |
| **Verubulin (MPC-6827; Azixa®)**  (An synthetic analogue of combretastatin) | - | Disruption of tumor vasculature by interrupting microtubule formation | Yes  NCT00394446  NCT00393965  NCT01285414  NCT00892931  NCT00609011 | Myrexis Inc. (now: Immune Pharmaceuticals) | Grossmann et al. 2012; Mahal et al. 2014 |
| **Cryptophycins** | Nostoc cyanobacteria | Interference with microtubule dynamics | No^$^ | - | Weiss et al. 2013; Weiss et al. 2017 |
| **Noscapine** (and its analogues) | *Papaver somniferum* | Interference with microtubule dynamics | Yes^β^  NCT00912899  NCT00183950 | Cougar Biotechnology, Inc. | Henary et al. 2014; Mishra et al. 2015 (for review: DeBono et al. 2015) |
| **Epothilones** | *Sorangium cellulosum* (a myxobacteria) | Stabilization of microtubules | Yes^±^ | - | Brogdon et al. 2014; Yu et al. 2013;Forli, 2014 |
| **Discodermolide** | Marine sponge (*Discodermia dissoluta*) | Stabilization of microtubules | No | - | ter Haar et al. 1996; von Schwarzenberg and Vollmar, 2013 |
| **Spongistatin 1** | Marine sponge (Spirastrella spinispirulifera | Inhibition of the phosphorylation activity of protein kinase Calpha (PKCalpha) | No | - | Rothmeier et al. 2009; von Schwarzenberg and Vollmar,2013 |
| **Anguidine (Diacetoxyscirpenol)** | *Fusarium* species | Inhibition of HIF-1 | Yes | - | Murphy et al. 1978; Choi et al. 2016 |
| **Fumagillin** (and its analogue TNP-470) | *Aspergillus fumigatus* | Disruption of tumor vasculature by targeting methionine aminopeptidase type 2 | Yes  NCT00038701  NCT00000763 | - | van Wijngaarden et al. 2010; Kornienko et al. 2015 |
| **Wortmannin** (and its analogue PX-866) | Fungus species | Inhibition of metastasis and angiogenesis via PI3K/Akt/NF-κB-mediated MMP-9 and IL-8 pathways | Yes  NCT01616199  NCT01259869  NCT01252628  NCT00726583  NCT01204099 | Cascadian Therapeutics Inc. | Li et al. 2012 (for review: Kornienko et al. 2015) |
| **Cytochalasin E** | Fungi and lichens | Inhibition of endothelial cell proliferation | No | - | Udagawa et al. 2000; Delebassée et al. 2017 (for review: Kornienko et al. 2015) |
| **Chaetocin** | *Chaetomium spp* and other fungi | Inhibition of endothelial cell proliferation and HIF-1α down-regulation | No | - | Lee et al. 2011; Isham et al. 2012; (for review: Kornienko et al. 2015) |
| **Epoxyquinol B** | Fungi | Inhibition of VEGFR2, EGFR, FGFR, and PDGFR | No | - | Kamiyama et al. 2008 |
| **Halichondrin B** and its analogue eribulin (Halaven™) | Marine sponges (*Halichondria* species) | Depolymerization of microtubules and tumor microenvironment modulation | Yes^@^ | Eisai Inc. | Cortes et al. 2011; Dybdal-Hargreaves et al. 2015; Ito et al. 2017 |
| **Embellistatin** | Marine fungi | Inhibition f microtubule polymerization | No | - | Jung et al. 2007 |
| **Taccalonolides** | *Tacca* species | Microtubule stabilization^κ^ | No | - | Risinger et al. 2013; Wang et al. 2017 (for review : Risinger et al. 2010; Li et al. 2014) |
| **Pseudolaric acid B** | *Pseudolarix* species | Inhibition of HIF-1α by promoting proteasome degradation | No | - | Li et al. 2004 (reviewed in Miao et al.2012) |
| **Sanguinarine** | *Sanguinaria canadensis* | Inhibition of VEGF | No | - | Dong et al. 2013; Xu et al. 2013 |
| **Apigenin** | Various plants | Modulation of TGF-β pathway | Yes  NCT03139227  NCT00609310 | - | Mirzoeva et al. 2014 |
| **Zerumbone** | *Zingiber* species | Blockade of NF –κB activity | No | - | Shamoto et al. 2014 (for review, see: Rahman et al. 2014) |
| **Deoxypodophyllotoxin** | *Podophyllum* species | Destabilization of microtubules | No | - | Jiang et al. 2013 |

^#^Verified using the NIH database (www.clinicaltrials.gov)

**^†^**Details about related clinical trials can be found in ClinicalTrials.gov at <https://www.clinicaltrials.gov/ct2/results?cond=&term=vadimezan&cntry=&state=&city=&dist>

^ǂ^Details about related clinical trials can be found in ClinicalTrials.gov at <https://www.clinicaltrials.gov/ct2/results?cond=&term=dolastatin+10+&cntry=&state=&city=&dist>

^¥^Details about related clinical trials can be found in ClinicalTrials.gov at <https://www.clinicaltrials.gov/ct2/results?cond=&term=lurbinectedin&cntry=&state=&city=&dist>

^§^Details about related clinical trials can be found in ClinicalTrials.gov at <https://www.clinicaltrials.gov/ct2/results?term=curcumin&cond=Cancer&Search=Apply&recrs=b&recrs=a&recrs=f&recrs=d&recrs=g&recrs=h&recrs=e&age_v=&gndr=&type=&rslt>

^$^Cryptophycin-52 was tested in phase II for schizophrenia but failed because of its high neurotoxicity.

^β^Terminated because of lack of efficacy.

^±^Details about related clinical trials can be found in ClinicalTrials.gov at <https://www.clinicaltrials.gov/ct2/results?term=Epothilones&cond=Cancer&Search=Apply&recrs=b&recrs=a&recrs=f&recrs=d&recrs=h&recrs=e&age_v=&gndr=&type=&rslt>

^@^Details about related clinical trials can be found in ClinicalTrials.gov at <https://www.clinicaltrials.gov/ct2/results?cond=&term=halichondrin&cntry=&state=&city=&dist>

^κ^Reviewed in two recent papers discussing the potential of microtubule-stabilizing and destabilizing agents from natural sources: Cao YN, Zheng LL, Wang D, et al. Recent advances in microtubule-stabilizing agents. Eur J Med Chem. 2018;143:806-828. doi: 10.1016/j.ejmech.2017.11.062. / Risinger AL, Peng J, Rohena CC, Aguilar HR, Frantz DE, Mooberry SL. The Bat Flower: a Source of Microtubule Destabilizing and Stabilizing Compounds with Synergistic Antiproliferative Actions. J Nat Prod. 2013;76(10):1923-1929. doi:10.1021/np4005079.

**References**

Belgiovine C, Bello E, Liguori M, et al. Lurbinectedin reduces tumour-associated macrophages and the inflammatory tumour microenvironment in preclinical models. Br J Cancer. 2017;117(5):628-638. doi:10.1038/bjc.2017.205.

Block KI, Gyllenhaal C, Lowe L, et al. A Broad-Spectrum Integrative Design for Cancer Prevention and Therapy. Semin Cancer Biol 2015;35(Suppl):S276-S304. doi:10.1016/j.semcancer.2015.09.007.

Brogdon CF, Lee FY, Canetta RM. Development of other microtubule-stabilizer families: the epothilones and their derivatives. Anticancer Drugs. 2014;25(5):599-609. doi: 10.1097/CAD.0000000000000071.

Buchanan CM, Shih JH, Astin JW, et al. DMXAA (Vadimezan, ASA404) is a multi-kinase inhibitor targeting VEGFR2 in particular. Clin Sci (Lond);122(10):449-57. doi: 10.1042/CS20110412.

Céspedes MV, Guillén MJ, López-Casas PP, et al. Lurbinectedin induces depletion of tumor-associated macrophages, an essential component of its in vivo synergism with gemcitabine, in pancreatic adenocarcinoma mouse models. Dis Model Mech. 2016;9(12):1461-1471. doi:10.1242/dmm.026369.

Choi Y-J, Shin H-W, Chun Y-S, Leutou AS, Son BW, Park J-W. Diacetoxyscirpenol as a new anticancer agent to target hypoxia-inducible factor 1. Oncotarget. 2016;7(38):62107-62122. doi:10.18632/oncotarget.11529.

Cortes J, O'Shaughnessy J, Loesch D, et al. Eribulin monotherapy versus treatment of physician's choice in patients with metastatic breast cancer (EMBRACE): a phase 3 open-label randomised study. Lancet. 2011;377(9769):914-23. doi: 10.1016/S0140-6736(11)60070-6.

DeBono A, Capuano B, Scammells PJ. Progress Toward the Development of Noscapine and Derivatives as Anticancer Agents. J Med Chem. 2015;58(15):5699-727. doi: 10.1021/jm501180v.

Delebassée S, Mambu L, Pinault E, et al. Cytochalasin E in the lichen Pleurosticta acetabulum. Anti-proliferative activity against human HT-29 colorectal cancer cells and quantitative variability. Fitoterapia. 2017;121:146-151. doi: 10.1016/j.fitote.2017.07.006.

Dong X, Zhang M, Wang K, et al. Sanguinarine Inhibits Vascular Endothelial Growth Factor Release by Generation of Reactive Oxygen Species in MCF-7 Human Mammary Adenocarcinoma Cells. Biomed Res Int. 2013;2013:517698. doi:10.1155/2013/517698.

Dybdal-Hargreaves NF, Risinger AL, Mooberry SL. Eribulin Mesylate: Mechanism of Action of a Unique Microtubule Targeting Agent. Clin Cancer Res. 2015;21(11):2445-2452. doi:10.1158/1078-0432.CCR-14-3252.

Elez ME, Tabernero J, Geary D, et al. First-in-human phase I study of Lurbinectedin (PM01183) in patients with advanced solid tumors. Clin Cancer Res. 2014;20(8):2205-14. doi: 10.1158/1078-0432.CCR-13-1880.

Fanale D, Bronte G, Passiglia F, et al. Stabilizing versus Destabilizing the Microtubules: A Double-Edge Sword for an Effective Cancer Treatment Option?. Anal Cell Pathol (Amsterdam). 2015;2015:690916. doi:10.1155/2015/690916.

Forli S. Epothilones: from discovery to clinical trials. Curr Top Med Chem. 2014;14(20):2312-2321.

Gajula PK, Asthana J, Panda D, et al. A synthetic dolastatin 10 analogue suppresses microtubule dynamics, inhibits cell proliferation, and induces apoptotic cell death. J Med Chem. 2013;56(6):2235-45. doi: 10.1021/jm3009629.

Grossmann KF, Colman H, Akerley WA, et al. Phase I trial of verubulin (MPC-6827) plus carboplatin in patients with relapsed glioblastoma multiforme. J Neurooncol. 2012;110(2):257-64. doi: 10.1007/s11060-012-0964-7.

Henary M, Narayana L, Ahad S, et al. Novel third-generation water-soluble noscapine analogs as superior microtubule-interfering agents with enhanced antiproliferative activity. Biochem Pharmacol. 2014;92(2):192-205. doi:10.1016/j.bcp.2014.07.020.

Howells LM, Berry DP, Elliott PJ, et al. Phase I randomised double-blind pilot study of micronized resveratrol (SRT501) in patients with hepatic metastases - safety, pharmacokinetics and pharmacodynamics. Cancer Prev Res (Philadelphia, Pa). 2011;4(9):1419-1425. doi:10.1158/1940-6207.CAPR-11-0148.

Hsu LC, Durrant DE, Huang CC, et al. Development of hemiasterlin derivatives as potential anticancer agents that inhibit tubulin polymerization and synergize with a stilbene tubulin inhibitor. Invest New Drugs. 2012;30(4):1379-88. doi: 10.1007/s10637-011-9702-9.

Huang L, Zhang Z, Zhang S, et al. Inhibitory action of Celastrol on hypoxia-mediated angiogenesis and metastasis via the HIF-1α pathway. Int J Mol Med. 2011;27(3):407-15. doi: 10.3892/ijmm.2011.600.

Isham CR, Tibodeau JD, Bossou AR, Merchan JR, Bible KC. The anticancer effects of chaetocin are independent of programmed cell death and hypoxia, and are associated with inhibition of endothelial cell proliferation. Br J Cancer. 2012;106(2):314-323. doi:10.1038/bjc.2011.522.

Ito K, Hamamichi S, Abe T, et al. Antitumor effects of eribulin depend on modulation of the tumor microenvironment by vascular remodeling in mouse models. Cancer Sci. 2017;108(11):2273-2280. doi:10.1111/cas.13392.

Jiang Z, Wu M, Miao J, et al. Deoxypodophyllotoxin exerts both anti-angiogenic and vascular disrupting effects. Int J Biochem Cell Biol. 2013;45(8):1710-9. doi: 10.1016/j.biocel.2013.04.030.

Jung HJ, Shim JS, Lee HB, et al. Embellistatin, a microtubule polymerization inhibitor, inhibits angiogenesis both in vitro and in vivo. Biochem Biophys Res Commun. 2007;353(2):376-80.

Kamiyama H, Kakeya H, Usui T, et al. Epoxyquinol B shows antiangiogenic and antitumor effects by inhibiting VEGFR2, EGFR, FGFR, and PDGFR. Oncol Res. 2008;17(1):11-21.

Kim SH, Hahm ER, Arlotti JA, et al. Withaferin A inhibits in vivo growth of breast cancer cells accelerated by Notch2 knockdown. Breast Cancer Res Treat. 2016;157(1):41-54. doi: 10.1007/s10549-016-3795-y.

Kornienko A, Evidente A, Vurro M, et al. Towards a Cancer Drug of Fungal Origin. Med Res Rev. 2015;35(5):937-967. doi:10.1002/med.21348.

Kuznetsov G, TenDyke K, Towle MJ, et al. Tubulin-based antimitotic mechanism of E7974, a novel analogue of the marine sponge natural product hemiasterlin. Mol Cancer Ther. 2009;8(10):2852-60. doi: 10.1158/1535-7163.MCT-09-0301.

Lee YM, Lim JH, Yoon H, Chun YS, Park JW. Antihepatoma activity of chaetocin due to deregulated splicing of hypoxia-inducible factor 1α pre-mRNA in mice and in vitro. Hepatology. 2011;53(1):171-80. doi: 10.1002/hep.24010.

Li J, Li F, Wang H, et al. Wortmannin reduces metastasis and angiogenesis of human breast cancer cells via nuclear factor-κB-dependent matrix metalloproteinase-9 and interleukin-8 pathways. J Int Med Res. 2012;40(3):867-76.

Li J, Risinger AL, Mooberry SL. Taccalonolide microtubule stabilizers. Bioorg Med Chem. 2014;22(18):5091-5096. doi:10.1016/j.bmc.2014.01.012.

Li MH, Miao ZH, Tan WF, et al. Pseudolaric acid B inhibits angiogenesis and reduces hypoxia-inducible factor 1alpha by promoting proteasome-mediated degradation. Clin Cancer Res. 2004;10(24):8266-74.

Mahal K, Resch M, Ficner R, et al. Effects of the tumor-vasculature-disrupting agent verubulin and two heteroaryl analogues on cancer cells, endothelial cells, and blood vessels. ChemMedChem. 2014;9(4):847-54. doi: 10.1002/cmdc.201300531.

Miao Z, Feng J, Ding J. Newly discovered angiogenesis inhibitors and their mechanisms of action. Acta Pharmacol Sin. 2012;33(9):1103-1111. doi:10.1038/aps.2012.97.

Mirzoeva S, Franzen CA, Pelling JC. Apigenin inhibits TGF-β-induced VEGF expression in human prostate carcinoma cells via a Smad2/3- and Src-dependent mechanism. Mol Carcinog. 2014;53(8):598-609. doi: 10.1002/mc.22005.

Mishra RC, Gundala SR, Karna P, et al. Design, Synthesis and Biological Evaluation of Di-substituted Noscapine Analogs as Potent and Microtubule-Targeted Anticancer Agents. Bioorg Med Chem Lett. 2015;25(10):2133-2140. doi:10.1016/j.bmcl.2015.03.076.

Mohan R, Hammers HJ, Bargagna-Mohan P, et al. Withaferin A is a potent inhibitor of angiogenesis. Angiogenesis. 2004;7(2):115-22.

Müller P, Martin K, Theurich S, et al. Microtubule-depolymerizing agents used in antibody-drug conjugates induce antitumor immunity by stimulation of dendritic cells. Cancer Immunol Res. 2014;2(8):741-55. doi: 10.1158/2326-6066.CIR-13-0198.

Murphy WK, Burgess MA, Valdivieso M, et al. Phase I clinical evaluation of anguidine. Cancer Treat Rep. 1978;62(10):1497-502.

Porcù E, Bortolozzi R, Basso G, Viola G. Recent advances in vascular disrupting agents in cancer therapy. Future Med Chem. 2014;6(13):1485-98. doi: 10.4155/fmc.14.104.

Rahman HS, Rasedee A, Yeap SK, et al. Biomedical Properties of a Natural Dietary Plant Metabolite, Zerumbone, in Cancer Therapy and Chemoprevention Trials. Biomed Res Int. 2014;2014:920742. doi:10.1155/2014/920742.

Reece KM, Richardson ED, Cook KM, et al. Epidithiodiketopiperazines (ETPs) exhibit in vitro antiangiogenic and in vivo antitumor activity by disrupting the HIF-1α/p300 complex in a preclinical model of prostate cancer. Mol Cancer. 2014;13:91. doi:10.1186/1476-4598-13-91.

Risinger A, Li J, Bennett M, et al. Taccalonolide binding to tubulin imparts microtubule stability and potent in vivo activity. Cancer Res. 2013;73(22):10.1158/0008-5472.CAN-13-1346. doi:10.1158/0008-5472.CAN-13-1346.

Risinger AL, Mooberry SL. Taccalonolides: Novel Microtubule Stabilizers with Clinical Potential. Cancer Lett 2010;291(1):14-19. doi:10.1016/j.canlet.2009.09.020.

Rothmeier AS, Ischenko I, Joore J, et al. Investigation of the marine compound spongistatin 1 links the inhibition of PKCalpha translocation to nonmitotic effects of tubulin antagonism in angiogenesis. FASEB J. 2009;23(4):1127-37. doi: 10.1096/fj.08-117127.

Shamoto T, Matsuo Y, Shibata T, et al. Zerumbone inhibits angiogenesis by blocking NF-κB activity in pancreatic cancer. Pancreas. 2014;43(3):396-404. doi: 10.1097/MPA.0000000000000039.

Shanmugam MK, Warrier S, Kumar AP, et al. Potential Role of Natural Compounds as Anti-Angiogenic Agents in Cancer. Curr Vasc Pharmacol. 2017;15(6):503-519. doi: 10.2174/1570161115666170713094319.

Siemann DW. The Unique Characteristics of Tumor Vasculature and Preclinical Evidence for its Selective Disruption by Tumor-Vascular Disrupting Agents. Cancer Treat Rev. 2011;37(1):63-74. doi:10.1016/j.ctrv.2010.05.001.

ter Haar E, Kowalski RJ, Hamel E, et al. Discodermolide, a cytotoxic marine agent that stabilizes microtubules more potently than taxol. Biochemistry. 1996;35(1):243-50.

Trapp V, Parmakhtiar B, Papazian V, Willmott L, Fruehauf JP. Anti-angiogenic effects of resveratrol mediated by decreased VEGF and increased TSP1 expression in melanoma-endothelial cell co-culture. Angiogenesis. 2010;13(4):305-315. doi:10.1007/s10456-010-9187-8.

Udagawa T, Yuan J, Panigrahy D, et al. Cytochalasin E, an epoxide containing Aspergillus-derived fungal metabolite, inhibits angiogenesis and tumor growth. J Pharmacol Exp Ther. 2000;294(2):421-7.

van Wijngaarden J, Snoeks TJ, van Beek E, et al. An in vitro model that can distinguish between effects on angiogenesis and on established vasculature: actions of TNP-470, marimastat and the tubulin-binding agent Ang-510. Biochem Biophys Res Commun. 2010;391(2):1161-5. doi: 10.1016/j.bbrc.2009.11.097.

von Schwarzenberg K, Vollmar AM. Targeting apoptosis pathways by natural compounds in cancer: marine compounds as lead structures and chemical tools for cancer therapy. Cancer Lett. 2013;332(2):295-303. doi: 10.1016/j.canlet.2010.07.004.

Wang Y, Yu Y, Li G-B, et al. Mechanism of microtubule stabilization by taccalonolide AJ. Nat Commun. 2017;8:15787. doi:10.1038/ncomms15787.

Weiss C, Figueras E, Borbely AN, et al. Cryptophycins: cytotoxic cyclodepsipeptides with potential for tumor targeting. J Pept Sci. 2017;23(7-8):514-531. doi: 10.1002/psc.3015.

Weiss C, Sammet B, Sewald N. Recent approaches for the synthesis of modified cryptophycins. Nat Prod Rep. 2013;30(7):924-40. doi: 10.1039/c3np70022d.

Xu J-Y, Meng Q-H, Chong Y, et al. Sanguinarine is a novel VEGF inhibitor involved in the suppression of angiogenesis and cell migration. Mol Clin Oncol. 2013;1(2):331-336. doi:10.3892/mco.2012.41.

Yu D, Pessino V, Kuei S, Valentine MT. Mechanical and functional properties of epothilone-stabilized microtubules. Cytoskeleton (Hoboken). 2013;70(2):74-84. doi: 10.1002/cm.21091.

Zhang JR, Lu F, Lu T, et al. Inactivation of FoxM1 transcription factor contributes to curcumin-induced inhibition of survival, angiogenesis, and chemosensitivity in acute myeloid leukemia cells. J Mol Med (Berl). 2014;92(12):1319-30. doi: 10.1007/s00109-014-1198-2.

**Supplementary Figures:**

**Supplementary Figure S1. Disposition of trabectedin clinical trials by phase number**

* Results of step 1: none of the experimental arms fulfills expectations and the study will not continue as a phase III (Source: [www.ClinicalTrials.gov](http://www.ClinicalTrials.gov) (assessed 14/08/2017)).

**Supplementary Figure S2. Disposition of OVA-301 phase III study**

**Supplementary Figure S3. Mechanism of action of combretastatin.** CA-4-P activates that RHO-GTP pathway leading to MLC phosphorylation, F-actin polymerization, stress-fibre formation, the assembly of focal adhesions and the dissociation of VE cadherin junctions. CA-4-P also activates SAPK2 that promotes ‘membrane blebbing’, characterized by cell rounding and the formation of surface blebs, misassembled focal adhesions and a dense F-actin peripheral band. Active ERK1/2 or MLCK attenuate the blebbing process. MCL-P, phosphorylated myosin light chain; MLCK, myosin light-chain kinase; p-ERK1/2, phosphorylated, active ERK1/2; SAPK2, stress-activated protein kinase 2. Reprinted by permission from Macmillan Publishers Ltd: [Nature Reviews Cancer] (Tozer GM, Kanthou C, Baguley BC. Disrupting tumour blood vessels. Nat Rev Cancer. 2005;5(6):423-35.), Copyright (2005).

**Supplementary Figure S4. Survival of patients with soft tissue sarcoma according to tumor tissue markers.** (A) These Kaplan–Meier curves illustrate (A, B) progression-free survival (PFS) and (C, D) overall survival (OS) according to excision repair
cross-complementation group 1 (ERCC1) status: (A) PFS and (C) OS are shown according to ERCC1 (rare allele) expression, and (B) PFS and (D) OS are shown according to toERCC1 (common allele) expression. (B) These Kaplan–Meier curves illustrate (top) progression-free survival (PFS) and (bottom) overall survival (OS) according to excision repair cross-complementation group 5 (ERCC5) status: (top left) PFS and (bottom left) OS are shown according to toERCC5 (common allele) expression, and (top right) PFS and (bottom right) OS are illustrated according to ERCC5 (rare allele) expression. Reprinted from Cancer, 117(15), Italiano et al. ERCC5/XPG, ERCC1, and BRCA1 gene status and clinical benefit of trabectedin in patients with soft tissue sarcoma, 3445-3456, Copyright (2011), with permission from John Wiley and Sons.

**Supplementary Figure S5. Survival of ovarian cancer patients treated with trabectedin.** (A) Analysis of progression-free survival (PFS) by independent radiology assessment of all measurable patients (primary endpoint). (B) Analysis of PFS for patients with platinum-sensitive disease. (C) Analysis of PFS for patients with platinum-resistant disease. (D) Interim analysis of overall survival (OS). HR, hazard ratio; PLD, pegylated liposomal doxorubicin. Reprinted with permission. © 2010 American Society of Clinical Oncology. All rights reserved. Monk BJ et al: J Clin Oncol 28(19);3107–3114.

**Supplementary Figure S1.**


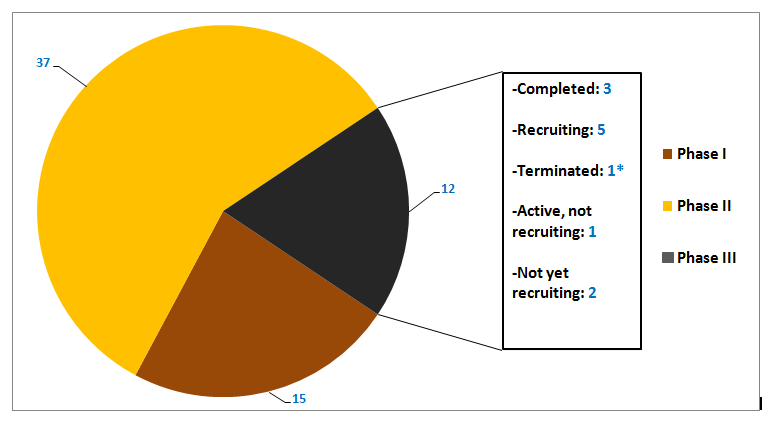


**Supplementary Figure S2.**

**Analyzed for PFS (N=317)**

**Confirmed complete response: N= 14**

**Analyzed for PFS (N=328)**

**Confirmed complete response: N= 24**

**Received travectedin + PLD (N=334)**

**Received PLD**

**(N=329)**

**Assigned to PLD**

**(N=335)**

**Assigned to trabectedin + PLD (N=337)**

**Randomization of 672 OC patients**

Excluded (N=3)

Excluded (N=6)

**Supplementary Figure S3.**

**
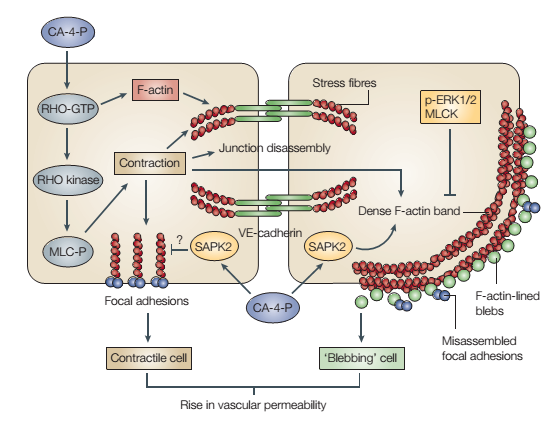
**

**Supplementary Figure S4.**


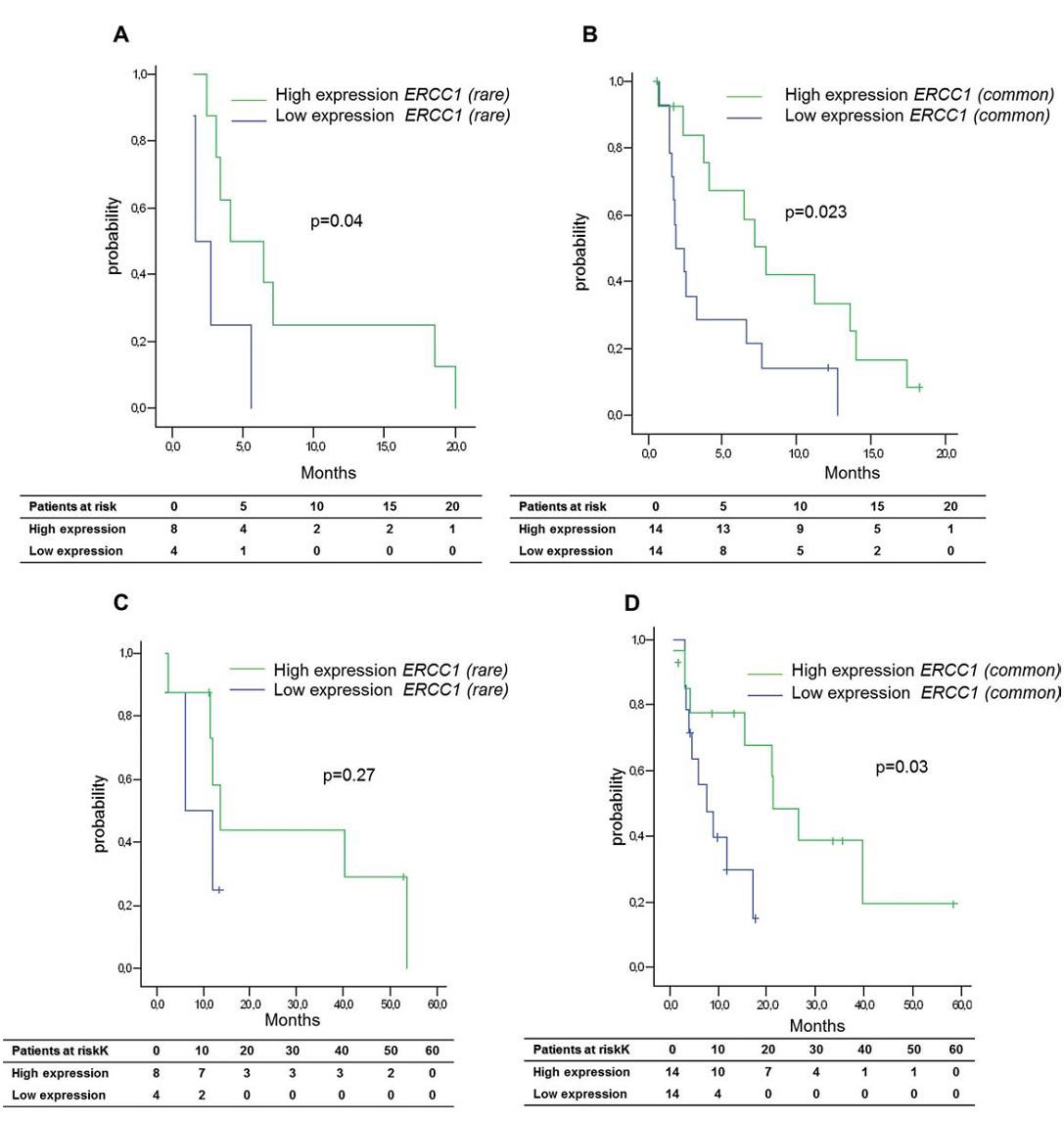


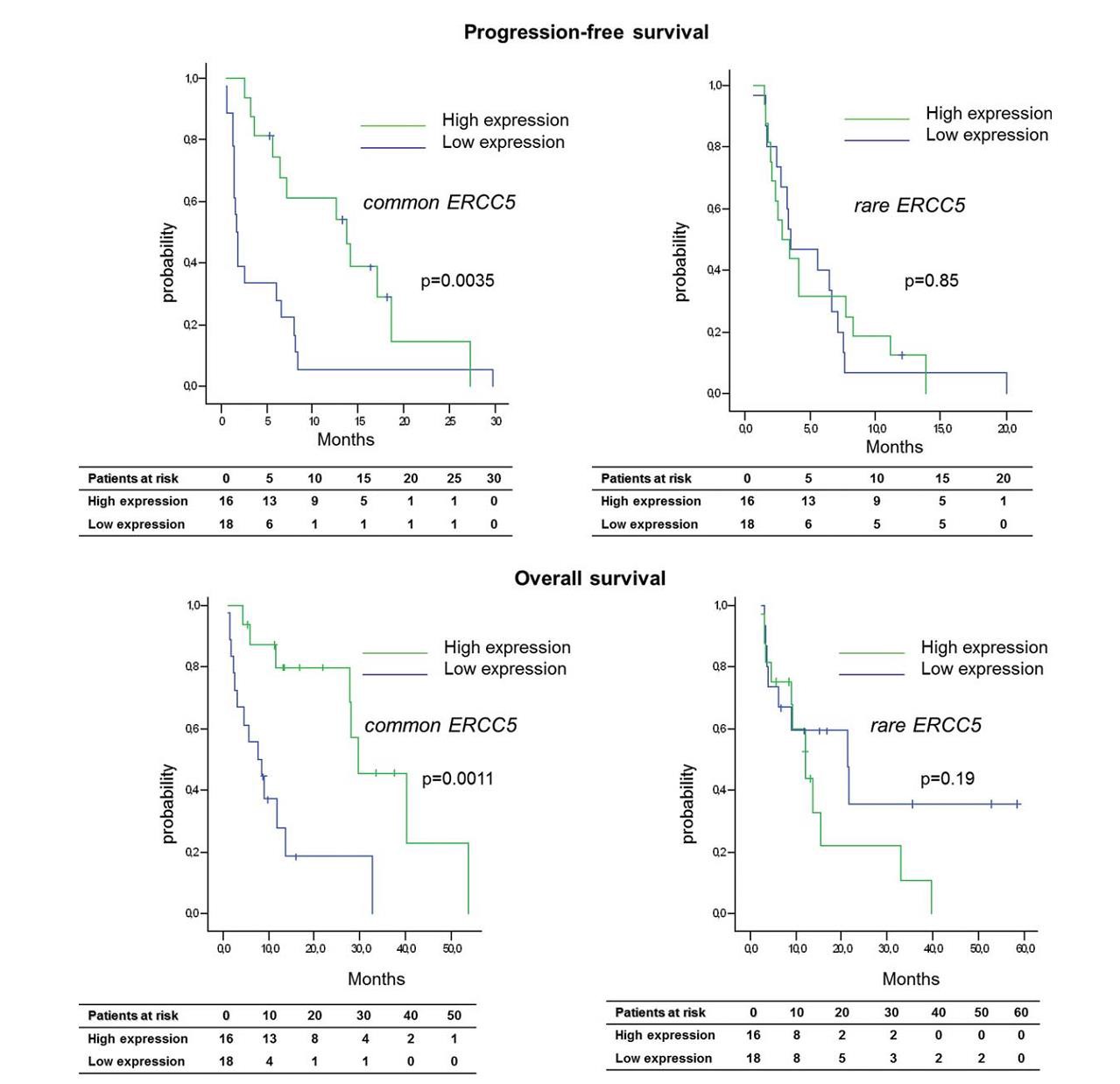


**Supplementary Figure S5.**

**
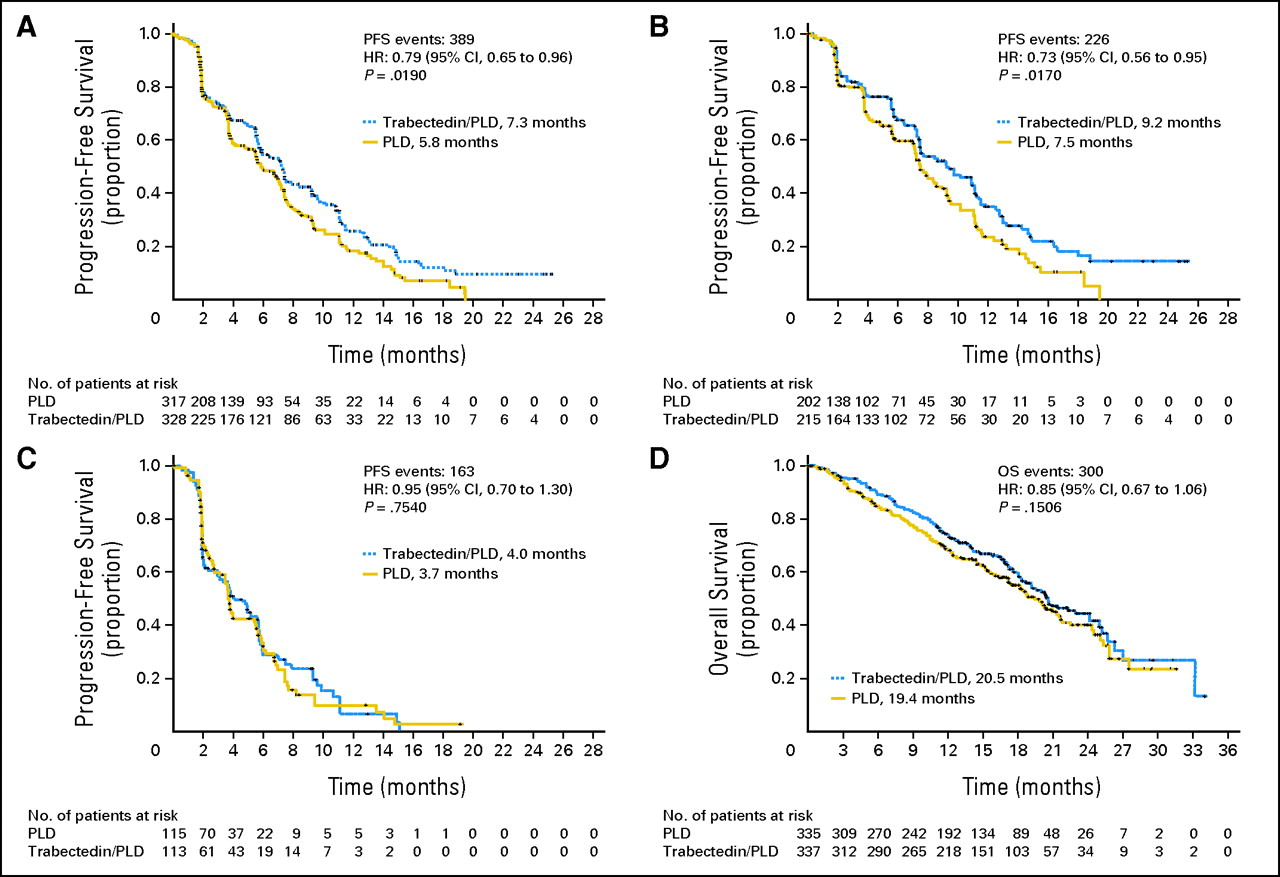
 \**

**Supplementary Boxes**

| **Supplementary Box S1. Useful list of *Medline*-indexed and highly accessed journals studying angiogenesis and related oncology clinical trials** | |
| --- | --- |
| **Angiogenesis** | <http://www.springer.com/biomed/cancer/journal/10456> |
| **Vascular Pharmacology** | <https://www.journals.elsevier.com/vascular-pharmacology/> |
| **Nature Reviews Cancer^(†)^** | <http://www.nature.com/nrc/index.html> |
| **Nature Reviews Drug Discovery^(†)^** | <http://www.nature.com/nrd/index.html> |
| **British Journal of Cancer** | <http://www.nature.com/bjc/index.html> |
| **European Journal of Cancer** | <https://www.journals.elsevier.com/european-journal-of-cancer/> |
| **Investigational New Drugs** | <http://www.springer.com/medicine/oncology/journal/10637> |
| **Drugs** | <http://www.springer.com/adis/journal/40265> |
| **BBA Reviews on Cancer^(†)^** | <https://www.journals.elsevier.com/bba-reviews-on-cancer> |
| **Cancer Treatment Reviews^(†)^** | <https://www.journals.elsevier.com/cancer-treatment-reviews/> |
| **Expert Review of Anticancer Therapy^(†)^** | <http://www.tandfonline.com/toc/iery20/current> |
| **Expert Opinion on Drug Discovery^(†)^** | <http://www.tandfonline.com/toc/iedc20/current> |
| **Expert Opinion on Therapeutic Targets^(†)^** | <http://www.tandfonline.com/toc/iett20/current> |
| **Expert Opinion on Pharmacotherapy^(†)^** | <http://www.tandfonline.com/toc/ieop20/current> |
| **Expert Opinion on Emerging Drugs^(†)^** | <http://www.tandfonline.com/toc/iemd20/current> |
| **Expert Opinion on Drug Safety^(†)^** | <http://www.tandfonline.com/toc/ieds20/current> |
| **Expert Opinion on Biological Therapy^(†)^** | <http://www.tandfonline.com/toc/iebt20/current> |
| **Expert Opinion on Orphan Drugs^(†)^** | <http://www.tandfonline.com/toc/ieod20/current> |
| **Expert Opinion on Investigational Drugs^(†)^** | <http://www.tandfonline.com/toc/ieid20/current> |
| **Cancer Discovery** | <http://cancerdiscovery.aacrjournals.org/> |
| **Molecular Cancer Therapeutics** | <http://mct.aacrjournals.org/> |
| **Seminars in Cancer Biology** | <https://www.journals.elsevier.com/seminars-in-cancer-biology/> |
| **(†) Journals publishing only review articles** | |

| **Supplementary Box S2. Additional useful reviews of particular interest** |
| --- |
| Gnanambal K ME, Lakshmipathy SV. **Dictyoceratidan poisons: Defined mark on microtubule-tubulin dynamics**. Life Sci. 2016;148:229-40. doi: 10.1016/j.lfs.2016.02.034.  Anjum K, Abbas SQ, Akhter N, et al. **Emerging biopharmaceuticals from bioactive peptides derived from marine organisms**. Chem Biol Drug Des. 2017;90(1):12-30. doi: 10.1111/cbdd.12925.  Kita M, Kigoshi H. **Marine natural products that interfere with multiple cytoskeletal protein interactions**. Nat Prod Rep. 2015;32(4):534-42. doi: 10.1039/c4np00129j.  Rodrigues T, Reker D, Schneider P, Schneider G. **Counting on natural products for drug design**. Nat Chem. 2016;8(6):531-41. doi: 10.1038/nchem.2479.  Harvey AL, Edrada-Ebel R, Quinn RJ. **The re-emergence of natural products for drug discovery in the genomics era**. Nat Rev Drug Discov. 2015;14(2):111-29. doi: 10.1038/nrd4510.  Koehn FE, Carter GT. **The evolving role of natural products in drug discovery**. Nat Rev Drug Discov. 2005;4(3):206-20. doi: 10.1038/nrd1657.  Wang Z, Dabrosin C, Yin X, et al. **Broad targeting of angiogenesis for cancer prevention and therapy**. Semin Cancer Biol 2015;35(Suppl):S224-S243. 10.1016/j.semcancer.2015.01.001  Liu Z, Xu P, Wu T, Zeng W. **Microtubule-targeting anticancer agents from marine natural substance**. Anticancer Agents Med Chem. 2014;14(3):409-17.  Newman DJ, Cragg GM. **Natural Products as Sources of New Drugs from 1981 to 2014**. J Nat Prod. 2016;79(3):629-61. doi: 10.1021/acs.jnatprod.5b01055.  Pye CR, Bertin MJ, Lokey RS, Gerwick WH, Linington RG. **Retrospective analysis of natural products provides insights for future discovery trends**. Proc Natl Acad Sci U S A. 2017;114(22):5601-5606. doi: 10.1073/pnas.1614680114.  Amin ARMR, Kucuk O, Khuri FR, Shin DM. **Perspectives for Cancer Prevention With Natural Compounds**. J Clin Oncol. 2009;27(16):2712-2725. doi:10.1200/JCO.2008.20.6235.  Shen B. **A New Golden Age of Natural Products Drug Discovery**. Cell. 2015;163(6):1297-1300. doi: 10.1016/j.cell.2015.11.031 |
